# Development of a core outcome set for studies on centralization of healthcare services

**DOI:** 10.1186/s12913-026-14861-z

**Published:** 2026-06-09

**Authors:** Stefanie Pfisterer-Heise, Julia Scharfe, Alexander Pachanov, Charlotte Mareike Kugler, Eni Shehu, Tim Mathes, René Mantke, Dawid Pieper

**Affiliations:** 1 Institute for Health Services and Health System Research, Center for Health Services Research Brandenburg, Brandenburg Medical School Theodor Fontane (MHB), Faculty of Health Sciences Brandenburg, Rüdersdorf bei Berlin, Germany; 2 Department of Medical Statistics, University Medical Center Göttingen, Göttingen, Germany; 3 Clinic for General and Visceral Surgery, University Hospital Brandenburg, Brandenburg an der Havel, Germany

**Keywords:** Centralization, Centralisation, Regionalization, Regionalisation, Core outcome set, Minimum volume standard, Volume-outcome relationship, Evidence synthesis, Health policy

## Abstract

**Background:**

Centralization defined as the reorganization of healthcare services into fewer specialized units serving a higher volume of patients is a strategy to improve the outcome quality of medical procedures. Studies on centralization, however, tend to measure and report different outcomes impeding evidence syntheses. To advance research on the effects of centralization, this study aimed at developing a core outcome set (COS) for studies on centralization of hospital procedures. The envisaged COS should be generic and include all relevant outcomes irrespective of available data or measurement instruments.

**Methods:**

For developing the COS, we conducted (1) a systematic review on the effects of minimum volume standards in hospitals, (2) a focus group study with patient representatives, (3) two interview studies with representatives of medical societies and statutory health insurance funds, (4) an online study with health services researchers, (5) the development of a longlist of outcomes and (6) a two-round Delphi study with all aforementioned participants. All interest-holder groups were based in Germany. The collected outcomes were categorized according to the Cochrane Effective Practice and Organisation of Care (EPOC) outcome taxonomy. A priori, we defined to include an outcome if ≥75% of all participants in a respective interest-holder group rated an outcome with 7–9 (critically important) on a 9-point Likert scale, and if this criterion was met by at least two interest-holder groups.

**Results:**

In total, 61 participants took part in the focus groups (*n* = 14), the interviews (*n* = 28) and the online survey (*n* = 19). Of these 36 and 34 participated in Delphi I and II, respectively. From the studies, 48 outcomes were derived and presented in Delphi I. Based on participants’ comments 45 outcomes were displayed in the Delphi II survey. The final COS comprises 27 outcomes in all EPOC outcome domains. Nine of these belong to the EPOC outcome domain “Outcomes related to the quality of care”.

**Conclusions:**

Future studies on centralization should measure and report outcomes from a broad range of outcome domains, including outcomes in the domain “Quality of care”. Instruments for measuring these outcomes need to be consented.

**Supplementary Information:**

The online version contains supplementary material available at 10.1186/s12913-026-14861-z.

## Introduction

Centralization is a health policy strategy, which denotes the reorganization of healthcare services into fewer specialized units serving a higher volume of patients [1]. It is based on the volume-outcome relationship stating that a higher volume of a particular intervention is associated with better outcome quality [2, 3]. This relationship has been shown for many different interventions and is grounded in evidence for the practice-makes-perfect hypothesis [4–8]. Hence, centralization, i.e. the concentration of cases, can be described as a quality improvement strategy, particularly with respect to the outcome quality of complex medical procedures commonly associated with high complication rates [9–12].

Oftentimes these medical interventions, for instance cancer surgery, transplantations and care for preterm infants, are centralized by introducing minimum volume standards [13–19]. These are normatively set minimum thresholds. Hospitals, which do not reach this minimum volume standard, are then no further authorized to perform a specific procedure, for example enforced by being no longer reimbursed. For patients, this may mean longer travel distances to treating hospitals, which can be particularly burdensome for patients from remote or rural areas [20, 21]. Still, there is extensive evidence in favor of centralization, particularly in reducing patient mortality, which has been shown for different interventions such as cancer surgery and neonatal care [18, 22, 23].

However, in studies on centralization methodological challenges remain. First, although centralization is a phenomenon on the health system level affecting many different groups of interest-holders, studies for instance tend to neglect outcomes directly affecting healthcare professionals such as training positions for surgeons [24]. Instead and in line with studies on the volume-outcome relationship, studies on centralization typically focus on patient-related outcomes, for example mortality [9, 22, 25–27]. Furthermore, studies on centralization tend to measure and report different outcomes, which makes it difficult to compare and synthesize study results. However, to be able to estimate the effects of centralization, it is first important to define the most important outcomes, taking into account outcomes on all levels. Second, the same outcomes should be measured and reported across studies.

A methodological tool to establish a consented collection of outcomes for a particular research phenomenon are so-called core outcome sets (COS). These are agreed standardized sets of outcomes that should be measured and reported as a minimum in all studies [28]. Core outcome sets were brought forward and are promoted by the COMET (Core Outcome Measures in Effectiveness Trials) Initiative with the idea of improving the standards of reporting and data synthesis [29]. While COS were originally intended to be developed for studies on specific diseases, today the scope of COS has been widened to different research phenomena, such as informed consent for surgery or work participation [30, 31].

With the aim of advancing research on centralization, this study set out to develop a core outcome set for future studies on centralization of healthcare services. A priori we defined that this COS should meet the following three criteria: 1. be generic for studies on centralization of health care services, i.e. exclude all outcomes with a focus on a specific disease, 2. include all relevant outcomes irrespective of whether data or measurement instruments are available at the time of the study, 3. adopt an international perspective. These criteria were determined so that future international studies may evaluate centralization of healthcare services on a meta, non-disease specific level. At the same time, this COS should guide future research with respect to the development of measurement instruments for core outcomes in studies on centralization.

## Methods

The study was registered with the COMET Initiative [32]. It adheres to the Core Outcome Set- STAndards for Development: The COS-STAD recommendations [33]. A priori, a study protocol was published [34]. The study received an ethics waiver by Brandenburg Medical School Theodor Fontane, Germany (Medizinische Hochschule Brandenburg, waiver no. E-01-20220630). For reporting, we follow the Core Outcome Set-Standards for Reporting: The COS-STAR Statement Checklist (Additional file 1) [35].

### Scope of the COS

The COS is generic for studies on the centralization of healthcare services. The target population are hospital patients of any sex, any age, irrespective of their condition or the hospital procedure received. The intervention is the centralization of healthcare services. Outcomes in all domains of the Cochrane Effective Practice and Organisation of Care (EPOC) outcome taxonomy were included [36] (Additional file 2). The COS is applicable to observational studies and experimental studies on centralization [34].

### Participants in the COS

We aimed at incorporating a broad range of perspectives on the topic of centralization including medicine, health economics and research. We therefore decided to invite the following four interest-holder groups to develop the COS: representatives of medical societies, representatives of statutory health insurance funds and health services researchers, all in Germany. Explicitly, we invited patient representatives as prior research has largely neglected patients’ perspectives [34, 37]. Eligibility criteria for all participants included: 1. being mandated by their respective organization and 2. full German proficiency.

To include further perspectives and discuss content-related matters, at the beginning of the project we established a project advisory board. This included clinicians, health services researchers and members of the Federal Joint Committee (Gemeinsamer Bundesausschuss, G-BA), the highest decision-making body of the joint self-government in the German healthcare system which is among others responsible for introducing minimum volume standards [38].

### Development process of the COS

**Fig. 1:**
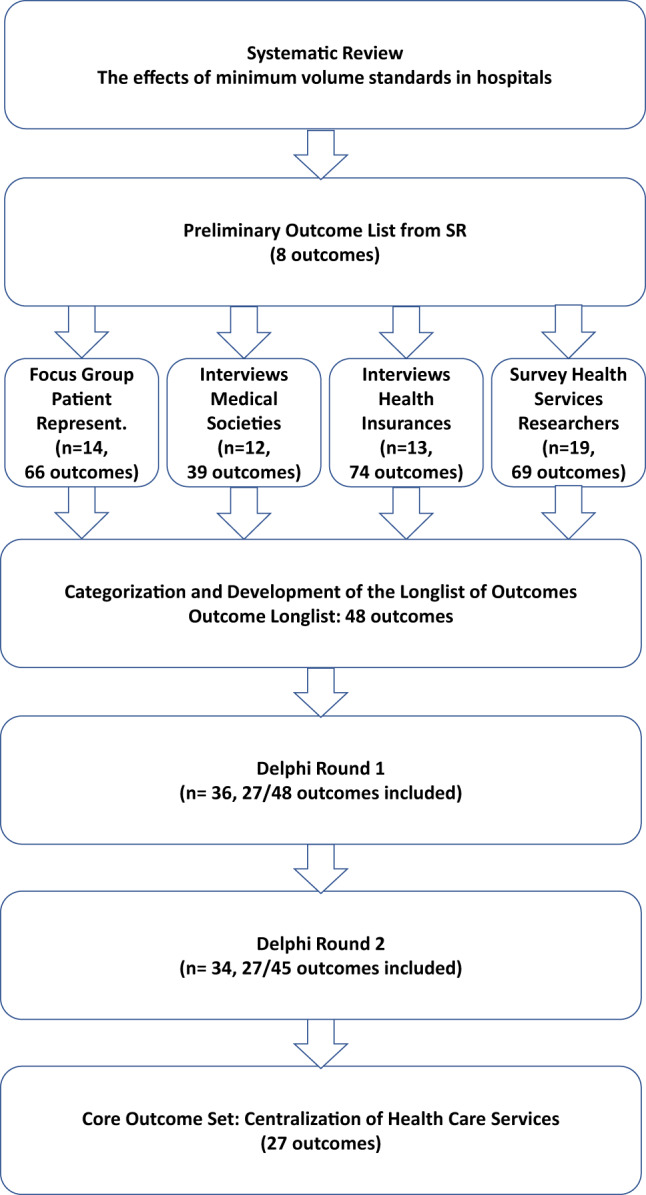
Development of a core outcome set for studies on centralization of healthcare services

The study ran from March 2022 to May 2024. The development of the COS for studies on the centralization of healthcare services comprised the following steps: 1. a systematic review (SR) on the effects of minimum volume standards in hospitals [39, 40], 2. a focus group study with representatives of patient advocacy groups in Germany, 3. two interview studies, one with representatives of medical societies and one with representatives of statutory health insurance funds, both in Germany, 4. an online survey with health services researchers in Germany, 5. the development of the longlist of outcomes including all outcomes generated by means of the aforementioned studies, 6. a two-round Online-Delphi study involving all the aforementioned interest-holder groups to prioritize the outcomes and to gain consensus on the outcomes to be included in the final COS (Fig. 1).

#### Systematic review

To gain an overview of outcomes measured and reported in studies on centralization, we conducted a SR on the effects of minimum volume standards in hospitals. A priori, the SR was registered with PROSPERO [41], and a protocol was published [40].

To be eligible for inclusion in the SR, studies must investigate the effects associated with minimum volume standards, defined as a minimum of specific healthcare procedures (or, specifically, treatments), usually defined by a cut-off, in a given timeframe and region [34]. Outcomes of interest comprised: (1) patient-related outcomes, (2) process-related outcomes, and (3) health system-related outcomes [40]. Studies with the following design were included: (cluster) randomized controlled trials ([C]RCTs), non-randomized controlled trials (nRCTs), controlled before-after studies (CBAs), and interrupted time-series studies (ITSs) [40]. There were no restrictions with respect to the regulator (e.g. state authority, regional authority, and professional association), the year of implementation, selected standards or cutoff points, and consequences in case of noncompliance (e.g., non-reimbursement for the performed procedure). No restrictions on language, publication date, or publication status were imposed. From the literature search for the SR a preliminary outcome list was derived.

#### Focus group study with patient representatives

For collecting additional outcomes, we planned a focus group study with 8 to 10 patient representatives, i.e. representatives of self-help groups and professional spokespeople. Eligible patient advocacy groups were identified based on the following criteria for indications: 1. minimum volume standards were in effect in Germany (at the time of the study this was the case for 10 procedures), 2. minimum volume standards might be introduced based on the announcements by the Federal Joint Committee [42], 3. minimum volume standards might be introduced based on international trends of centralization [19]. We then searched the internet for patient advocacy groups in Germany referring to these criteria. Furthermore, we recruited representatives of patient advocacy groups on a broader, non-disease specific level, such as for example the Federal Syndicate of Patient Interest Groups (BAGP). We approached all relevant patient advocacy groups via email including information material on the study background as well as a preliminary list of outcomes from the literature search for the SR (Additional file 3). In addition, representatives of patient advocacy groups were offered a compensation of 150 € each for taking part in the focus group and the Delphi study.

With the aim of collecting relevant outcomes in studies on the centralization of healthcare services, we developed a focus group guide. To be as accessible as possible, this included key questions on the advantages and disadvantages of centralization, i.e. the effects of centralization (see Additional file 4). Prior to participation, all participants gave their written informed consent. Furthermore, they were offered a testing session with Cisco WebEx (San José, USA) which one patient representative took advantage off. The focus groups were facilitated by the first author (SPH) on 16 September and 13 October 2022 respectively. Due to the geographical distances between participants, both focus groups were conducted via the online-meeting-tool WebEx. Both group discussions were recorded with the online tool WebEx and then transcribed by an external agency.

For analyzing the qualitative data, two researchers (SPH, JS) independently extracted relevant outcomes using MAXQDA 2022. Then, both researchers checked the list of the respective other for completeness and accuracy. The lists were merged, duplicates removed and discrepancies resolved by discussion.

#### Interview studies with representatives of medical societies and statutory health insurance funds

To include medical and health-economic perspectives, we conducted semi-structured interviews with representatives of the medical societies and of statutory health insurance funds in Germany, planning for 12 to 15 interviews with each interest-holder group. For recruiting eligible medical societies, we followed the list of indications affected or possibly affected by minimum volume standards (see above). Based on this list, we searched the Register of the Association of Scientific Medical Societies, (Arbeitsgemeinschaft Wissenschaftlicher Medizinischer Fachgesellschaften, AWMF), the umbrella organization of medical societies in Germany [43]. We contacted all eligible medical societies via email, asking for an interview with their president at the time.

In case of the statutory health insurance funds we contacted the 25 biggest in terms of insured persons [44]. Furthermore, we searched the internet for representatives of statutory health insurance funds who had published presentations on centralization and/ or minimum volume standards or who had been cited in an interview on the topic. We approached all representatives of statutory health insurance funds via email.

Prior to conducting the interviews, the focus group guide for patient representatives (see above) was adapted according to the two other interest-holder groups (Additional file 5). Furthermore, to verify the appropriateness of the interview guide, we shared it with a clinic director and chair for general and visceral surgery (RM). All other procedures and settings (i.e. provision of information material and online facilitation) were equal to those for patient representatives outlined above. The interviews were conducted by the first author (SPH) between September and December 2022. Three researchers (SPH, JS, AP) then extracted relevant outcomes (for examples see Additional file 6).

#### Online survey with health services researchers

To include health services researchers’ perspectives, we designed an online survey using LimeSurvey, Version 5.6.65 (Additional file 7). In the introduction, this online survey listed some outcomes in different EPOC outcome domains. All of these examples were based on the results of the literature search as part of the systematic review and served to stimulate reflection on outcomes in different domains in studies on centralization. Participants were then asked to state outcomes that they deemed important in studies on the centralization of healthcare services using free text boxes. The length of the survey was approximately ten minutes. We aimed at recruiting 20 to 25 participants.

The link to the online survey was sent out on 5 December 2022 with a short text in the Newsletter of the German Network for Health Services Research (Deutsches Netzwerk Versorgungsforschung, DNVF). The DNVF is an interdisciplinary network open to all institutions, working groups and scientists involved in the improvement of health and healthcare from a scientific, practical or health policy point of view [45].

#### Developing the longlist of outcomes

A research team consisting of four health services researchers (SPH, JS, AP, DP; 2 male, 2 female; one sociologist, one psychologist, two public health experts, one of them being a certified nurse) developed the longlist of outcomes. To this end, all extracted outcomes from the focus group discussions, the interviews and the online survey were collected, merged into one list and duplicates removed. Content and wording of all outcomes were discussed until consensus was reached. Lastly, outcomes were categorized according to the EPOC outcome taxonomy [36]. The final longlist contained outcomes irrespective of whether measurement instruments or data sources were known. Due to the generic scope of the COS, outcomes with a focus on a specific disease, e.g. degree of tumor removal for cancer, were deleted from the longlist.

#### Delphi study

To achieve consensus among all interest-holder groups regarding the importance of outcomes, a two-round Delphi study was employed using LimeSurvey, Version 5.6.65 (Additional file 8).

All participants of the focus groups, the interview studies and the online survey who had given their written consent to be invited to the Delphi study and had provided their email address were invited to both parts of the Delphi study. As participation was anonymous the invitation to Delphi II was sent regardless of the fact, whether the respective interest-holder had taken part in Delphi I.

Before sending the invitations to the Delphi study, the survey was pilot-tested by a patient representative. The invitations to Delphi I were then sent out between 22 and 27 June 2023 as personalized emails including the link to the Delphi survey and a pdf file with a list of all the outcomes in the longlist presented in Delphi I. Reminder phone calls and emails were sent out or respectively carried out between 5 and 9 July 2023. The invitation for Delphi II was sent out in one email addressed to all interest-holders on 18 September 2023. This email included the link to Delphi II and a pdf file with the list of commentaries on the outcomes presented in Delphi I.

In both Delphi rounds, participants were asked to rate each of the presented outcomes on a nine-point Likert scale from 1–3 (not important), 4–6 (important but not critical) and 7–9 (critically important) [46]. An option “no response” was provided for each outcome. Furthermore, a free text field for commenting on one’s rating was presented below each outcome. To prevent order effects, outcome domains were presented in randomized order and within each domain outcomes were presented in randomized order [47]. Furthermore, based on the reasoning of the COMET Initiative, which clearly distinguishes between the “what” to measure and the “how” to measure, all outcomes were presented without definitions or operationalizations [46]. At the beginning and at the end of the survey, participants were invited to comment on the instruction of the Delphi study and on the survey as a whole. At the end of Delphi I, participants were invited to add further outcomes not yet included in the survey. Furthermore, in Delphi I and II they were asked for their age and gender and in case of representatives of medical societies for the respective medical society (Delphi II).

The COMET Initiative does not recommend specific criteria for consensus. Hence, the following consensus criteria were determined a priori based on the COMET key consideration “that there should be good representation from key stakeholder groups with qualified experts who have a deep understanding of the issues” [46]. Furthermore, the threshold was set based on the median threshold in Delphi studies as shown in a systematic review [48]. Specifically, we a priori determined that 1. every interest-holder group is weighed equally, 2. the minimum number of participants in each interest-holder group is five, 3. consensus to include an outcome in the respective outcome set is when ≥75% of all participants in the respective interest-holder group rated 7–9 (critically important) for an outcome, 4. the overall COS consists of outcomes included by at least two interest-holder groups. This was shared with all Delphi study participants before starting the survey. Furthermore, for defining how outcomes were revised between the Delphi rounds, we determined that at least two comments needed to suggest a revision. If only one participant suggested a revision, outcomes could be revised with justified exception, for example in cases that an outcome was clearly misunderstood.

## Results

### Systematic review

In total, 12,878 potentially relevant studies on the effects of minimum volume standards were retrieved through electronic databases and trial registries, while additional 821 records were discovered through reference lists of included articles, forward citation searches, websites, and by contacting authors directly. After examining 192 full-text reports, 9 studies were subsequently included in the SR [11, 12, 21, 22, 25, 49–52], the results of which are submitted elsewhere [39].

The studies focused on various surgical procedures subject to minimum volume standards, including lung cancer surgeries, bariatric surgeries, esophagectomies, liver resections, gastrectomies, abdominal aortic aneurysm repairs and pancreatic resections. The following outcomes were measured and reported in the included studies: mortality (operative, 30 days), short-time mortality rate, travel distance, number of complications, number of reoperations, number of procedures, number of procedures in high volume/ designated hospitals, length of stay. The preliminary outcome list from the SR on the effects of minimum volume standards in hospitals thus included eight outcomes.

### Focus group study with patient representatives

Two focus groups with 14 representatives of patient advocacy groups (7 participants in each focus group) were conducted (see Table 3 for sociodemographic characteristics and participation rates of all studies within the development of the core outcome set). The indications and procedures covered by participants ranged from care for premature babies to kidney diseases, heart diseases and several kinds of cancers. One indication, more precisely leukemia, was represented by two patient advocacy groups. Prostate cancer was represented by two representatives from the same patient advocacy group, albeit different self-help groups, taking part in one focus group each. Both focus groups took up the entire planned time of 90 minutes. A list with 66 outcomes was compiled from both focus groups with patient representatives.

### Interviews with representatives of medical societies

In total, 12 interviews with 13 representatives of medical societies in Germany were conducted (Table 3). The medical indications and procedures covered ranged from complex surgeries on the esophagus and pancreas, surgical treatment of breast cancer and care for premature babies. Interviewee positions included current or future president of the respective medical society; one interview partner served as a secretary of the medical society. Interviews lasted on average 40 minutes (SD = 11.5). A list with 39 outcomes was compiled from the interviews with representatives of the medical societies in Germany.

### Interviews with representatives of statutory health insurance funds

In total, 13 interviews with 15 representatives of statutory health insurance funds were conducted (Table 3). These included the three largest German statutory health insurance funds according to the number of insured persons. Furthermore, a representative of the Federal Association of Company Health Insurance Funds (BKK Dachverband) and of the Federal Association of Local Health Care Funds (AOK Bundesverband) were interviewed. Positions of interviewees included for example CEO and Head of Inpatient Care and Care Management. Interviews lasted on average 37 minutes (SD = 10.48). In total, 74 outcomes were extracted from the interviews with representatives of statutory health insurance funds in Germany.

### Online survey with health services researchers

A priori, we had defined to invite the interest-holder group of health services researchers by means of the DNVF newsletter. To address a more targeted sample, we post-hoc decided to specifically invite health services researchers who had previously published in the field of centralization. We searched PubMed for articles on centralization and/ or minimum volume standards published by research teams in Germany. A list with 142 health services researchers in Germany was derived. We invited these researchers with a personalized email on 23 January 2023. No further implementation methods such as pre-contacting potential participants, using other types of surveys or reminder phone calls were employed [53]. Following this invitation, a total of 19 health services researchers (13%) participated in the survey (Table 3), 10 of these with more than 10 years-experience in health services research; 15 with more than 5 years-experience. 14 researchers (75%) stated that they had previously carried out research on centralization; 17 (89%) had done research in the field of minimum volume standards. Self-reported expertise on the topic of centralization of health care services on a five-point Likert scale with 5 = Very high expertise was on average 3.5 (SD = 1.14); self-reported expertise on the topic of centralization was 3.6 (SD = 1.1). From the online survey with health services researchers, a list with 69 outcomes was compiled.

### Development of the longlist of outcomes

Based on the aforementioned studies we collected four lists including the following number of outcomes: 66 outcomes from the focus groups with patient representatives, 39 outcomes from the interviews with representatives of the medical societies, 74 outcomes from the interviews with representatives of statutory health insurance funds, 69 outcomes from the survey with health services researchers. As a first step, we combined these lists and cleaned the resulting list of duplicates. In case that outcomes were conceptually close (e.g. 30-day-mortality and 1-year mortality, distance to therapy and distance to aftercare), we merged these outcomes. Lastly, we categorized all outcomes according to the EPOC scheme. In cases, in which an outcome could have been assigned to several outcome domains, as in case of the outcome *Morbidity (complications, health complaints)* (Potential outcome domains: “Patient-related outcomes” or “Adverse effects”), we discussed the categorization within the research team until consensus was reached. Furthermore, as there were outcomes that could not be categorized into any of the EPOC domains, for example *Shifts in the provision of hospital services*, we decided to introduce an outcome domain “Diverse Outcomes”. In total, the longlist of outcomes included 48 outcomes in all EPOC outcome domains (Table 1) [36].

**Table 1:** Longlist of outcomes as presented in Delphi I

Outcome domains	Outcomes
Patient related outcomes	Quality of life
	Morbidity (complications, health complaints)
	Mortality
	Satisfaction with healthcare
Outcomes related to the quality of care	Adherence to guidelines, clinical pathways or concepts
	Outpatient care (quality)
	Relationship between patient and members of the treatment team (quality)
	Diagnostics (quality)
	Indication (quality)
	Complication management (quality)
	Multi-professional care/ multi-disciplinarity of care
	Emergency care (quality)
	Degree of structuring of care pathways
	Therapy success/ treatment quality
Outcomes related to the utilization of health services	Visits to outpatient physicians (number)
	Surgical procedures (number)
	Inpatient treatments (number, duration)
Outcomes related to access to healthcare	Waiting time
	Digitilization/ telemedicine
	Proximity of care to place of residence (distance, travel time)
	Treatment/ therapy options (number/quality)
Outcomes related to the use of health care resources	Outpatient/ regional providers (number)
	Hospital beds (number)
	Costs for the healthcare system
	Cost efficiency of the healthcare system
	Hospitals (number)
	Staffing levels
	Technical equipment of the inpatient providers
Outcomes related to the use of non-health care resources	Visitors for patients
	Accommodation options for family/ relatives close to hospital
Health care provider outcomes	Employee workload
	Employee turnover
	Employee job satisfaction
	Training positions for junior physicians (quality, number)
	Routine of members of the treatment team (number of cases)
	Routine of surgeons (number of cases)
Outcomes related to equity of healthcare	Health care equity independent of the region
	Health care equity independent of patients’ sociodemographic factors
Adverse effects or harms	Revisions
	Adverse events
	Readmissions
	Wound infections
Diverse outcomes	Shifts in the provision of hospital services
	Co-operations between health service providers
	Psychological safety of the population with respect to healthcare
	Outpatient care physicians’ knowledge about healthcare
	Patients’ knowledge about healthcare
	Transparency (e.g. of treatment quality and clinical pathways)

### Delphi study

In Delphi I, these 48 outcomes were presented in 10 outcome domains. As with *Training positions for junior doctors* there was only one outcome referring to employment, we did not include the EPOC outcome domain “Social outcomes” and instead decided to present *Training positions for junior doctors* in the domain “Health care provider outcomes”.

36 participants took part in Delphi I: 12 patient representatives, 6 representatives of the medical societies, 8 representatives of statutory health insurance funds, 19 health services researchers (Table 3). Based on participants’ comments 11 of 48 outcomes were revised (Table 2) with no further outcomes added.

**Table 2:** Revision of outcomes: Delphi I to Delphi II

Delphi I: Outcomes	Delphi I: Comments (number of)	Delphi II: Outcomes
Quality of life	Comments suggesting to relate the outcome to health (2).	Health-related quality of life
Outpatient care (quality)	Questions of understanding (2).	Outpatient care in ambulatory settings (quality)
Degree of structuring of care pathways	Questions of understanding (2).	Clinical pathways (quality)
Therapy success/ treatment quality	Comments that treatment quality and therapy success are not congruent (2).	Individual treatment success
Waiting time	Questions of understanding (2).	Waiting time from indication to treatment/ surgical procedure
Treatment/ therapy options (number, quality)	Comments that outcome was not understood (2)	Access to treatment/ therapy options (number, quality)
Routine of members of the treatment team (number of cases)	Comment indicating that outcome was misunderstood (1).	Routine/ experience of the treatment team (number of cases)
Routine of surgeons (number of cases)	Comment indicating that outcome was misunderstood (1).	Routine/ experience of surgeons (number of cases)
Revisions, Adverse effects, Readmissions, Wound infections	Comments that outcome was not understood (2). Suggestions to introduce “Adverse effects” as top term(2).	Adverse effects (revisions, readmissions, wound infections)
Co-operations between health service providers	Comment introducing “networking” between health service providers (1).	Co-operations/ networking between health service providers
Psychological safety of the population with respect to healthcare	Comments that outcome was not understood (4).	Population’s trust in healthcare

Accordingly, in Delphi II 45 outcomes were presented including the ratings of each interest-holder group in Delphi I as recommended by the COMET Initiative (for outcomes presented in Delphi II see Additional file 9) [46]. 34 participants took part in Delphi II, 9 patient representatives, 7 representatives of the medical societies, 9 representatives of statutory health insurance funds, 9 health services researchers (Table 3).

**Table 3:** Sociodemographic characteristics and participation rates

	Focus Groups Patient representatives (n = 2)	Interviews Medical Societies (n = 12)	Interviews Statutory Health Insurance Funds (n = 13)	Online survey Health Services Researchers	Delphi I	Delphi II
Participants n (%)	14 (-)	13 (-)	15 (-)	19* (-)	36 (63)	34 (60)
Patient representatives	14 (-)	0 (-)	0 (-)	0 (-)	12 (86)	9 (64)
Medical societies representatives	0 (-)	13 (-)	0 (-)	0 (-)	6 (46)	7 (54)
Statutory health insurances representatives	0 (-)	0 (-)	15 (-)	0 (-)	8 (53)	9 (60)
Health services researchers	0 (-)	0 (-)	0 (-)	19 (-)	10 (67)	9 (60)
Gender						
Male	7 (50)	13 (100)	11 (73)	10 (53)	20 (56)	20 (59)
Female	7 (50)	0 (0)	4 (27)	9 (47)	16 (44)	14 (41)
Mean age (SD)	59 (10.8)	58 (4)	48 (8.3)	43 (10.7)	51 (12)	51 (11.1)

*Only 15 health services researchers provided their email-address for the Delphi study

In Delphi II, the following number of outcomes were voted with 7 to 9 (critically important) on a 9-point-Likert scale: patient representatives 33 outcomes, representatives of statutory health insurance funds 21 outcomes, representatives of medical societies 23 outcomes, health services researchers 24 outcomes. We also calculated mean values as well as minimum and maximum scores of outcomes (Additional file 9) with *Emergency care (quality)* achieving the highest possible average score *M* = 9 (SD = 0) by patient representatives (max statutory health insurance funds *Routine/ experience of surgeons M* = 8.56, SD = 0.53; max medical societies *Staffing levels M* = 8.29, SD = 0.49; max health services researchers *Emergency care (quality) M* = 8.13, SD = 0.99)

In total 27 outcomes of 45 presented outcomes were rated into the overall COS with 75% or more participants in two or more interest-holder groups voting the respective outcome with 7 to 9 (critically important) (Table 4).

**Table 4:** Final COS for studies on centralization of healthcare services categorized according to EPOC

Outcome domains	Outcomes
Patient-related outcomes	Health-related quality of life
	Morbidity (complications, health complaints)
	Mortality
Outcomes related to the quality of care	Adherence to guidelines, clinical pathways or concepts
	Outpatient care in ambulatory settings (quality)
	Diagnostics (quality)
	Indication (quality)
	Complication management (quality)
	Multi-professional care/ multi-disciplinarity of care
	Emergency care (quality)
	Clinical pathways (quality)
	Individual treatment success
Outcomes related to utilization, coverage or access	Surgical procedures (number)
	Access to treatment/ therapy options (number/quality)
Outcomes related to resource use	Staffing levels
	Technical equipment of the inpatient providers
Health care provider outcomes	Employee workload
	Employee job satisfaction
	Routine/ experience of members of the treatment team (number of cases)
	Routine/ experience of surgeons (number of cases)
Social outcomes	Training positions for junior physicians (quality, number)
Equity	Health care equity independent of the region
	Health care equity independent of patients’ sociodemographic factors
Adverse effects or harms	Adverse effects (revisions, readmissions, wound infections)
Diverse outcomes	Co-operation/ networking between health service providers
	Outpatient care physicians’ knowledge about healthcare
	Transparency (e.g. of treatment quality and clinical pathways)

## Discussion

This study aimed at developing a generic COS for studies on the centralization of healthcare services. To this end, perspectives of the following four interest-holder groups were included: patient representatives, representatives of the medical societies and of statutory health insurance funds, health services researchers, all in Germany. A COS including 27 outcomes resulted from this work.

One reason for this considerably high number of outcomes in comparison to six as the median number of outcomes in COS for research might be the scope of the COS [54]. In contrast to other COS which typically focus on a specific disease, the proposed COS is generic for studies on centralization, so on a broader, more general level. Based on this rationale, we decided to define outcomes on a rather low level of granularity [55]. For instance, the outcome *Emergency care (quality)* itself consists of different single indicators, i.e. waiting time. Accordingly, choosing a rather low level of granularity might have resulted in a lack of specificity of the included outcomes. At the same time, we propose that the resulting COS has a high level of sensitivity in the sense of including all outcomes, which are relevant for interest-holders.

Also, a priori we decided to include outcomes irrespective of data and measurement instruments so far being available. Outcomes might thus be part of the COS which would not have been included, in case that the COS was based on obtainable instruments and data. However, participants from the beginning knew the inclusion criteria into the COS. Furthermore, in Delphi II participants could see the ratings of each interest-holder group and thus be aware of the consequences of their ratings. Still, 27 outcomes were rated as “critically important” by at least two interest-holder groups and thus into the final COS.

When looking at the COS, it is noticeable that outcomes in all EPOC outcome domains are included. Nine of these 27 outcomes belong to the outcome domain “Outcomes related to the quality of care”. Both facts are remarkable, as studies in the field of centralization so far focus on patient-related outcomes and adverse effects, while neglecting outcomes in other outcome domains [12, 21, 25, 49, 51]. Another frequently reported outcome in studies on centralization is *Distance to hospital* [12, 21]. However, *Distance to hospital* was not voted into the COS by any of the interest-holder groups. This is a surprising result, as almost any centralization intervention is accompanied by longer distances for patients. It is even more surprising, as *Emergency care (quality)*, for which *Distance to the hospital* is usually an important aspect, achieved very high scores.

We assume the following reasons for the outcome *Distance to hospital* not being included in the final COS: First, our focus group sample consisted of patient representatives that were already affected by serious illnesses. As several of these patient representatives stated, they accepted very long distances to hospitals and even followed their treating physicians to receive the treatment they deemed best. This is in accordance with a systematic review showing that patients are willing to travel longer to lower their surgical risk [56]. Second, we interviewed representatives of the German medical societies who all but one worked in university hospitals, i.e. in metropolitan areas, and who might thus be less affected themselves by centralization interventions.

One might argue that the outcome *Distance to hospital* would have been included in the COS, if our sample had comprised representatives of smaller hospitals as well as people from the general population. However, during the entire course of the study we emphasized that we were interviewing physicians as representatives of their respective medical societies and not of their respective hospitals. Furthermore, we chose patient representatives and not patients or people from the general population, as due to the broad focus of the project it was more important that they had adequate knowledge of the healthcare system instead of the underlying disease [34].

Lastly, as stated in our a priori defined criteria, the developed COS should adopt an international perspective. Still, the development of the COS for studies on centralization is based exclusively on interest-holders’ perspectives from Germany. Hence, we cannot rule out that the COS on centralization includes or excludes outcomes based on specific views in Germany. For instance, interest-holders in countries with greater distances might have voted the outcome “Distance to hospital” into the COS.

### Limitations

Although we fully followed the COS-STAD recommendations with respect to the sample of this COS study, there are limitations. First, our sample does not cover all medical subject areas with respect to minimum volume standards in patient advocacy groups and medical societies alike thus not reaching congruity between both perspectives. Furthermore, despite our best recruiting efforts, we were not able to recruit representatives of a medical society in the fields of intensive care or pediatric surgery.

Also, we included a sample solely from Germany. On the one hand, this was due to funding restrictions. On the other hand, conducting this COS study internationally would probably have been impeded by language barriers, particularly of patient representatives in Germany. By contrast, conducting this COS study solely in Germany, facilitated the approach to present the outcomes without definitions, as in this case we could assume a similar understanding among the experts. Lastly, the subsample sizes of the interest-holder groups in the Delphi study were between 6 and 12 participants which is below the recommended subsample size of 20 to 30 for moderate replicability levels [57].

### Implications for future research

Our study bears the following implications for future research: So far, studies in the field of centralization mainly measure and report patient-related outcomes which can be explained by two main reasons: 1. Data on patient-related outcomes, for instance, mortality or reoperations might be more easily available than data on for example quality of care 2. Patient-related outcomes and adverse effects might be more easily measurable than outcomes in other outcome domains.

In contrast to this, the COS for studies on the centralization of health care services shows that outcomes from all EPOC domains including the domain “Quality of care” need to be acknowledged. As these outcomes are more complex, consensus regarding their measurement should be reached. So far, oftentimes different frameworks for these outcomes exist, which is the case for instance for the outcome *Emergency care (quality)* [58–61]. In case, that no measurement instruments exist, these need to be developed and consented.

To discuss measurement and data availability of the outcomes derived, we conducted a workshop with experts in Germany. The resulting expert recommendations will be published elsewhere. Furthermore, the COS for studies on the centralization of health care services should be employed in other countries, first to evaluate its applicability with respect to the included outcomes. For instance, the health system in Germany is characterized by a clear separation between ambulatory care and inpatient care [62]. Accordingly, researchers in other countries need to evaluate to what extent the included outcomes “Outpatient care (Quality)” and “Outpatient care physicians’ knowledge about healthcare” can be applied in their specific healthcare systems. Lastly, potential differences particularly in data availability need to be elicited.

## Conclusion

The core outcome set for studies on the centralization of inpatient healthcare services includes outcomes in all EPOC outcome domains. Future studies should take this into account and consider outcomes beyond patient-related outcomes. Further research should develop measurement recommendations for quality-related outcomes in particular.

## Electronic supplementary material

Below is the link to the electronic supplementary material.

Supplementary Material 1

Supplementary Material 2

Supplementary Material 3

Supplementary Material 4

Supplementary Material 5

Supplementary Material 6

Supplementary Material 7

Supplementary Material 8

Supplementary Material 9

## Data Availability

The datasets used and/ or analyzed during the current study are available from the corresponding author on reasonable request.
