# Supplementary material for: Development of a core outcome set for studies on centralization of healthcare services
Source: BMC Health Serv Res. 2026 Jun 9;26:810. doi: 10.1186/s12913-026-14861-z (PMC13255221; doi:10.1186/s12913-026-14861-z)
Supplement: Supplementary file 1 — Supplementary Material 1 [file 12913_2026_14861_MOESM1_ESM.pdf]

Core Outcome Set-STandards for Reporting: The COS-STAR Statement Checklist

SECTION/TOPIC	ITEM No.	CHECKLIST ITEM	REPORTED ON PAGE NUMBER
TITLE/ABSTRACT			
Title	1a	Identify in the title that the paper reports the development of a COS	1
Abstract	1b	Provide a structured summary	2-3
INTRODUCTION			
Background and Objectives	2a	Describe the background and explain the rationale for developing the COS.	4-5
	2b	Describe the specific objectives with reference to developing a COS.	5
Scope	3a	Describe the health condition(s) and population(s) covered by the COS.	6
	3b	Describe the intervention(s) covered by the COS.	6
	3c	Describe the setting(s) in which the COS is to be applied.	6
METHODS			
Protocol/Registry Entry	4	Indicate where the COS development protocol can be accessed, if available, and/or the study registration details.	5
Participants	5	Describe the rationale for stakeholder groups involved in the COS development process, eligibility criteria for participants from each group, and a description of how the individuals involved were identified.	6
Information Sources	6a	Describe the information sources used to identify an initial list of outcomes.	7
	6b	Describe how outcomes were dropped/combined, with reasons (if applicable).	10-11
Consensus Process	7	Describe how the consensus process was undertaken.	11-12
Outcome Scoring	8	Describe how outcomes were scored and how scores were summarised.	12
Consensus Definition	9a	Describe the consensus definition.	12
	9b	Describe the procedure for determining how outcomes were included or excluded from consideration during the consensus process.	11-12
Ethics and Consent	10	Provide a statement regarding the ethics and consent issues for the study.	5, 8, 24
RESULTS			
Protocol Deviations	11	Describe any changes from the protocol (if applicable), with reasons, and describe what impact these changes have on the results.	14
Participants	12	Present data on the number and relevant characteristics of the people involved at all stages of COS development.	Table 3

Outcomes	13a	List all outcomes considered at the start of the consensus process.	Table 1
	13b	Describe any new outcomes introduced and any outcomes dropped, with reasons, during the consensus process.	Table 2
COS	14	List the outcomes in the final COS.	Table 4
DISCUSSION			
Limitations	15	Discuss any limitations in the COS development process.	22
Conclusions	16	Provide an interpretation of the final COS in the context of other evidence, and implications for future research.	20-22
OTHER INFORMATION			
Funding	17	Describe sources of funding/role of funders.	24
Conflicts of Interest	18	Describe any conflicts of interest within the study team and how these were managed.	24

From: Kirkham JJ, Gorst S, Altman DG, Blazeby JM, Clarke M, Devane D, et al. (2016) Core Outcome Set–STAndards for Reporting: The COS-STAR Statement. PLoS Med 13(10): e1002148. https://doi.org/10.1371/journal.pmed.1002148
