# Supplementary material for: Development of a core outcome set for studies on centralization of healthcare services
Source: BMC Health Serv Res. 2026 Jun 9;26:810. doi: 10.1186/s12913-026-14861-z (PMC13255221; doi:10.1186/s12913-026-14861-z)
Supplement: Supplementary file 2 — Supplementary Material 2 [file 12913_2026_14861_MOESM2_ESM.pdf]

What outcomes should be reported in
Cochrane Effective Practice and Organisation of Care (EPOC) reviews?

Primary (main) outcomes

- Primary outcomes for EPOC reviews of health systems interventions should reflect those outcomes that are most important to the people who will be affected and that are critical or important to people making decisions. These outcomes normally should be included in a Summary of Findings table.
- We suggest referring to these as the '**main outcomes**'.
- Consideration should be given to including outcomes in each of the categories listed below.
- We suggest categorising outcomes using the categories listed below, although specific outcome measures will vary across studies and reviews.
- **Impacts on equity** (i.e. differential impacts on disadvantaged populations) should be considered for all outcomes. (See Equity considerations in EPOC reviews)
- **Adverse effects** should always be considered. (See Taking account of adverse effects in EPOC reviews)

1. Patient outcomes, including:
   - Health status and wellbeing, including:
     - Physical health and treatment outcomes: mortality, morbidity, surrogate physiological measures
     - Psychological health: psychological well being
     - Psychosocial outcomes: quality of life, social activities
   - Health behaviour, e.g. adherence to treatment or care plans, health care seeking behaviour

2. Quality of care, including:
   - Adherence to recommended practice or guidelines, e.g. extent to which health care providers gave specific advice, delivered specific interventions, followed referral guidelines

3. Utilisation, coverage or access, including:
   - Utilisation of services, e.g. of birthing facilities; length of stay in a facility
   - Coverage, e.g. proportion of children immunized or women who received antenatal care; enrolment to insurance programmes
   - Access to services, e.g. waiting times to see a doctor; recruitment and retention of health care providers

4. Resource use, including:
   - Healthcare resources, e.g. human resources/time, consumable supplies, buildings, equipment
   - Non-healthcare resources, e.g. transportation to healthcare facilities, social services
   - Patient and informal caregiver time[1]

---

[1] For further guidance, see: Chapter 15 (Incorporating economics evidence) of the Cochrane Handbook for Systematic Reviews of Interventions; and EPOC summary guidance on incorporating economic evidence in EPOC reviews.

Suggested citation: Cochrane Effective Practice and Organisation of Care (EPOC). [Title]. EPOC Resources for review authors, 2017. epoc.cochrane.org/resources/epoc-resources-review-authors (accessed DD Month YYYY)

5. Health care provider outcomes, including:
    - Workload
    - Work morale
    - Stress, burnout, sick leave

6. Social outcomes, including
    - Community empowerment or participation
    - Poverty measures
    - Employment
    - Education

7. Equity (differential effects across advantaged and disadvantaged populations): this needs to be considered for all of the other outcomes on this list

8. Adverse effects or harms, including adverse effects on:
    - Health or health behaviours (e.g. e.g. sepsis, the need for caesarean section)
    - Utilisation, coverage or access
    - Quality of care
    - Resource use
    - Health care providers (e.g. increased attrition, increased workload)
    - Social outcomes
    - Equity (i.e. increased inequities)
    - Clinical adverse effects, e.g. sepsis, the need for caesarean section

Secondary outcomes

- These are outcomes that may be of interest, but are less important than the primary outcomes. They are not critical or important to the people who will be affected or decision makers.
- They may indirectly reflect important outcomes (i.e. serve as surrogate outcome measures) or help to explain how or why an intervention did or might have an impact on primary outcomes.
- Review authors should specify whether studies that *only* report secondary outcomes will be included in the review.

1. Knowledge

2. Attitudes

3. Performance in a test situation

4. Satisfaction, including:
    - Healthcare recipients'
    - Providers'
    - Other stakeholders'
