# Supplementary material for: Development of a core outcome set for studies on centralization of healthcare services
Source: BMC Health Serv Res. 2026 Jun 9;26:810. doi: 10.1186/s12913-026-14861-z (PMC13255221; doi:10.1186/s12913-026-14861-z)
Supplement: Supplementary file 3 — Supplementary Material 3 [file 12913_2026_14861_MOESM3_ESM.pdf]

CONTACT

FAKULTÄT FÜR GESUNDHEITSWISSENSCHAFTEN BRANDENBURG

MHB MEDIZINISCHE HOCHSCHULE BRANDENBURG

Projekt lead

Prof. Dr. Dawid Pieper
Head of Institute for Health Services and Health System Research (IVGF)
and Center for Health Services Research (ZVF-BB)
Brandenburg Medical School (MHB)
Faculty of Health Sciences Brandenburg (FGW)

Project team

Stefanie Pfisterer-Heise (M.Sc.)
Institute for Health Services and Health System Research, MHB/FGW, ZVF-BB

Julia Scharfe (MPH)
Institute for Health Services and Health System Research, MHB/FGW, ZVF-BB

Registration and enquiries

Stefanie Pfisterer-Heise
Institute for Health Services and Health System Research, MHB/FGW, ZVF-BB
stefanie.heise@mhb-fontane.de, Phone: 033638-83995

MHB ZVF-BB Zentrum für Versorgungsforschung Brandenburg

Centralization of inpatient healthcare services

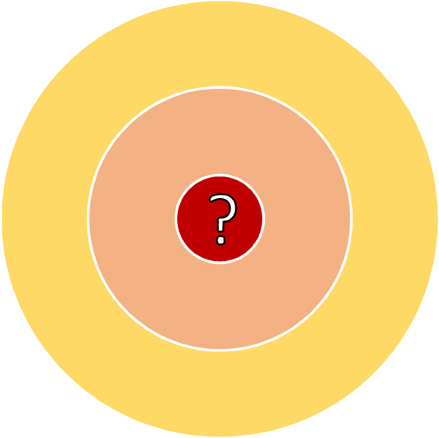

Information material on the research project MIVOS –

The effects of minimum volume standards in hospitals

Bundesministerium für Bildung und Forschung

(FKZ 01KG2107)

Dear madams and sirs, dear patient representatives,

Numerous studies are currently investigating the effects of centralizing inpatient healthcare services and the effects of introducing minimum volume standards. Often, different outcomes are reported in the published studies, whereby we understand these outcomes to be target values that can potentially be influenced by centralization or minimum volume standards. For example, one study reports that centralization had a negative effect on patient travel times, while another study reports that centralization had a positive effect on adherence to treatment guidelines.

The varying research and reporting of outcomes is a problem in that the effects of centralization can then no longer be synthesized or compared with each other. The aim of this research project is therefore to develop a **core outcome set (COS) for studies on the centralization of inpatient healthcare services**, whereby a set in this context is defined as several related similar or complementary items (such as knife, fork and spoon or jack, queen, king, ace). This core outcome set should then be measured and reported in all studies on centralization.

In preparation for our group discussion, we would like to present possible outcomes below that could be relevant in studies on the centralization of inpatient healthcare services. The target parameters listed here are neither exhaustive nor are they subject to judgement. The list presented is merely intended as food for thought and a basis for discussion in the focus groups. Based on your expertise and personal experience, you will no doubt find further relevant outcomes important.

Thank you in advance for your preparation and participation!

Outcomes that directly affect patients

- **Health-related quality of life** with its somatic, social and psychological dimensions;
- **Patient compliance**, i.e. the extent to which patients adhere to treatment plans;
- **Morbidity**, i.e. the complaints and complications of diseases, such as wound infections, post-operative haemorrhage, pneumonia, pulmonary embolism, etc.;
- **Readmission** to hospital with possible r**evision** surgery;
- **Hospital mortality**, i.e. deaths in hospital that are related in time to the treatment in question;
- **All-cause mortality**, ie. deaths that occur with a defined period of time after the treatment in question;

Outcomes related to access to treatment and the actual treatment

- **Travelling time** from the patient’s place of residence to hospital;
- **Waiting time** from the suspected diagnosis to the therapy/ intervention, i.e. the time that elapses between the first statement of suspicion of the specific disease by the attending physician to the start of the therapy actually based on the disease;
- **Adherence of the therapy to the clinical guidelines**;
- **Length of stay in hospital**;

Outcomes that affect healthservice providers

- **Amount of work** involved;
- **Physical and mental strain** on healthcare professionals;
