# Supplementary material for: Development of a core outcome set for studies on centralization of healthcare services
Source: BMC Health Serv Res. 2026 Jun 9;26:810. doi: 10.1186/s12913-026-14861-z (PMC13255221; doi:10.1186/s12913-026-14861-z)
Supplement: Supplementary file 4 — Supplementary Material 4 [file 12913_2026_14861_MOESM4_ESM.pdf]

Focus group guide "Centralization of inpatient healthcare services" (own translation to English)
Patient representatives

General outline	Specifying questions	Sustaining and guiding questions
**Introduction**		
Introduction	We would like to welcome you to our group discussion today. Thank you for taking the time to talk to us about the centralization of inpatient healthcare services. My name is Stefanie Pfisterer-Heise and I am a research associate at the Institute for Health Services and Health Systems Research at Medical School Brandenburg. Together with my colleague Julia Scharfe I am working here in the project MIVOS – The effects of minimum volume standards in hospitals, which is funded by the Federal Ministry of Education and Research.	
Project background	As part of our project, we are investigating which outcomes are important in research on the centralization of inpatient healthcare services and should therefore be measured in future studies. These can be outcomes such as mortality and morbidity, but also guideline adherence or the amount of work required on the part of service providers.	
Project aim	Our project aim is to develop a so-called Core Outcome Set for research on the centralization of inpatient healthcare services and to make it available to researchers. While developing the Core Outcome Set, it is important to us to involve all interest holders and, in particular, hear the opinions of patient representatives.<br>We have invited you because, as patient representatives, you have a direct connection to the topic of centralization of inpatient healthcare services and your opinions and experiences are important to us. We would therefore like to talk with you today about your experiences with centralization on the one hand and the advantages and disadvantages of centralizing inpatient healthcare services on the other hand. What is important in this discussion is that there are no right or wrong answers and you probably have different views. Please do not hesitate to let us know your point of view and your experiences, even if they differ from those of the other participants.	
Data protection	You have already given your informed consent to the recording of this group discussion in advance. Thank you very much for this. The results of this group discussion will of course be published anonymously, i.e. it won't be possible to draw any conclusions about you or your organizations. Please remember that we are just as interested in critical as in positive comments, as they help us to improve the quality of our research.	
Structure of group discussion	The group discussion will be structured in such a way that I will first ask questions about the advantages and disadvantages of centralizing inpatient healthcare services and then go into the topic of minimum volumes.	

Focus group guide “Centralization of inpatient healthcare services” (own translation to English)
Patient representatives

**General outline**	**Specifying questions**	**Sustaining and guiding questions**
Technical instruction<br><br>Questions	In due time we will start our group discussion. Before we start, I have two more comments on this digital format. Please think about muting yourself while not speaking, so that we can maintain the sound quality on a high level. Furthermore before speaking we would kindly like to ask you to virtually raise your hand. We would like to ensure that you can all contribute to the discussion. To do this in WebEx you find a small button saying „Raise hand“ or „Lower hand“.<br>Are there any more questions regarding the process or the IT? *Wait for comments*. If this is not the case, we will start our group discussion now.	

Start recording		
Personal information		
Opening question	First, I would like to suggest a short introductory round. Please tell us your name, your age and which organization you represent in this group discussion.	
Introduction/ Transitional question: **Today’s discussion is about the centralization of inpatient healthcare services. By this we mean that inpatient healthcare services are restructured in such a way that they are provided by fewer specialized providers with a higher patient volume. In which context have you come into contact with the topic “centralization of inpatient healthcare services“ in your function as a patient representative?**		
	To what extent are patients that you represent affected by the topic of centralization?<br>To what extend have you come into contact with the topic in other contexts, for example as a patient?	Could you tell us more about this?<br>Could you please give an example?<br>What do you mean specifically?
**Key question: In your opinion, what are the advantages and disadvantages of centralizing inpatient healthcare services for patient care? Please provide an explanation for your answer.**		
Clinical pathway<br><br>Follow-up care<br>Treatment quality	In your experience, what are the advantages and disadvantages for the patient’s pathway from symptoms to diagnosis to treatment?<br>What are the effects on follow-up care?<br>In your opinion, how does treatment quality change?	Could you tell us more about this?<br>Could you please give an example?<br>What do you mean specifically?
Key question: In your opinion, what are the advantages and disadvantages of centralization for patients?		
Patients<br><br>Relatives	What effects do you expect, for example, from the sometimes longer travel times for patients?<br>To what extent do you expect effects on relatives?	Could you tell us more about this?<br>Could you please give an example?

		What do you mean specifically?
***Key question: In your opinion, what other advantages and disadvantages does the centralization of inpatient healthcare services have? Please think about all interest holders and all areas of the healthcare system*.**		
Physicians and other medical professions in the healthcare system	In your opinion, to what extent do you expect effects for physicians and other medical professions?<br>What other advantages and disadvantages do you expect for the healthcare system?	Could you tell us more about this?<br>Could you please give an example?<br>What do you mean specifically?
***Key question*: As a means of centralizing inpatient healthcare services so-called minimum volume standards have been introduced in Germany. This means that cut-offs for a minimum number of a specific procedure were defined. Reaching this minimum volume standard is then a necessary prerequisite that a hospital is allowed to perform this procedure in the future. In which context have you come into contact with the topic of minimum volume standards?**		
	To what extent are patients that you represent affected by minimum volume standards?<br>To what extent have you come into contact with the topic in other contexts?	Could you tell us more about this?<br>Could you please give an example?<br>What do you mean specifically?
**Key question: In your opinion, what advantages and disadvantages are important in the context of minimum volume standards? Please think about all interest holders and all areas of the healthcare system.**		
		Could you tell us more about this?<br>Could you please give an example?<br>What do you mean specifically?
Summary	**Summary of the facilitator**	
Conclusion	Would anyone like to add something that he or she so far had not the opportunity to say? Did we forget anything important regarding today’s topics „centralisation of inpatient healthcare services“ or „minimum volume standards?	
**STOP recording**		
Contact in case of questions<br><br>Announcement of Delphi study	Can I contact you again in case that we have further questions?<br><br>After analyzing the group discussions, we will compile all outcomes obtained and have them rated with regard to their importance in a Delphi process. We would be very happy if you participated in the Delphi study. We will send you a link to your e-mail address provided.	
