# Supplementary material for: Development of a core outcome set for studies on centralization of healthcare services
Source: BMC Health Serv Res. 2026 Jun 9;26:810. doi: 10.1186/s12913-026-14861-z (PMC13255221; doi:10.1186/s12913-026-14861-z)
Supplement: Supplementary file 5 — Supplementary Material 5 [file 12913_2026_14861_MOESM5_ESM.pdf]

Interview guide “Centralization of inpatient healthcare services” (own translation)
Representatives of statutory health insurance funds

General outline	Specifying questions	Sustaining and guiding questions
**Introduction**		
Presentation<br>Project background	My name is (name, position, Institute for Health Services and Health Systems Research).<br>Today’s interview is about the centralization of inpatient healthcare services. By this we mean that inpatient healthcare services are restructured in such a way that they are provided by fewer specialized providers with a higher patient volume. Specifically, as part of our project, we are investigating which outcomes are important in research on the centralization of inpatient healthcare services and should therefore be measured in future studies. These can be outcomes such as mortality and morbidity, but also guideline adherence or the amount of work required on the part of service providers.	
Project aim	Our project aim is to develop a so-called Core Outcome Set for research on the centralization of inpatient healthcare services and to make it available to researchers.<br>We have invited you because, as a representative of a statutory health insurance fund, you have a direct connection to the topic of centralization of inpatient healthcare services and your opinions and experiences are important to us. We would therefore like to talk with you today about your experiences with centralization on the one hand and the advantages and disadvantages of centralizing inpatient healthcare services on the other.	
Skip questions/ Stop interview	You have already given your informed consent to the recording of this interview in advance. Thank you very much for this. During the interview, you always have the right to skip questions or stop the interview. The results will of course be published anonymously, i.e. it won’t be possible to draw any conclusions about you or your organization. Please remember that we are just as interested in critical as in positive comments, as they help us to improve the quality of our research.	
Structure of the interview<br>Questions	The interview will be structured in such a way that I will first ask questions about the advantages and disadvantages of centralizing inpatient healthcare services and then go into the topic of minimum volumes. Before we start the interview, do you have any questions?	

Interview guide “Centralization of inpatient healthcare services” (own translation)
Representatives of statutory health insurance funds

**START**		
Personal information		
Opening question	What exactly is your professional position? How old are you? Which statutory health insurance fund do you represent today in this interview?	
Key question: **In which context have you come into contact with the topic “centralization of inpatient healthcare services” in your capacity as a representative of a statutory health insurance fund?**		
	To what extent is your statutory health insurance fund affected by the topic of centralization?<br>To what extent have you come into contact with the topic in other contexts?	Could you tell me more about this?<br>Could you please give an example?<br>What do you mean specifically?
Key question**: In your opinion, what are the advantages and disadvantages of centralizing inpatient healthcare services for patient care? Please provide an explanation for your answer.**		
Clinical pathway<br><br>Follow-up care<br>Treatment quality	In your experience, what are the advantages and disadvantages for the patient’s pathway from symptoms to diagnosis to treatment?<br>What are the effects on follow-up care?<br>In your opinion, to what extent/ how does treatment quality change?	Could you tell me more about this?<br>Could you please give an example?<br>What do you mean specifically?
Key question: In your opinion, what are the advantages and disadvantages of centralization for your statutory health insurance fund?		
Structure and work processes<br><br>Policyholders	What general effects do you expect for the structure and work processes of statutory health insurance funds in Germany?<br>What effects will there be for your policyholders?	Could you tell me more about this?<br>Could you please give an example?<br>What do you mean specifically?
Key question: In your opinion, what other advantages and disadvantages does the centralization of inpatient healthcare services have? Please think about all interest holders and all areas of the healthcare system.		
Patients<br><br>Relatives<br>Healthcare system	What effects do you expect, for example, from the sometimes longer travel times for patients?<br>To what extent do you expect this to affect relatives?<br>What other advantages and disadvantages do you expect for the healthcare system?	Could you tell me more about this?<br>Could you please give an example?<br>What do you mean specifically?
Key question**: As a means of centralizing inpatient healthcare services minimum volume standards have been introduced in Germany. In which context have you come into contact with the topic of minimum volume standards?**		

	To what extent is your statutory health insurance fund affected by minimum volume standards?<br>To what extent have you come into contact with the topic in other contexts?	Could you tell me more about this?<br>Could you please give an example?<br>What do you mean specifically?
***Key question*: In your opinion, what advantages and disadvantages are important in the context of minimum volume standards? Please think about all interest holders and all areas of the healthcare system.**		
		Can you tell me more about this?<br>Could you please give an example?<br>What do you mean specifically?
Conclusion	**Is there anything else you would like to add to our conversation?**	
**STOP**		
Contact in case of questions<br><br>Announcement of Delphi study	Can I contact you again in case that further questions arise?<br><br>After analyzing all interviews, we will compile all outcomes obtained and have them rated with regard to their importance in a Delphi process. We would be very happy if you participated in the Delphi study. We will send you a link to your e-mail address provided.	
