# Supplementary material for: Development of a core outcome set for studies on centralization of healthcare services
Source: BMC Health Serv Res. 2026 Jun 9;26:810. doi: 10.1186/s12913-026-14861-z (PMC13255221; doi:10.1186/s12913-026-14861-z)
Supplement: Supplementary file 6 — Supplementary Material 6 [file 12913_2026_14861_MOESM6_ESM.pdf]

Example outcomes based on participants‘ quotes

Study	Quotes	Outcomes
**Patient organisation**	*“We would actually like to have a concept implemented, and Switzerland is always a bit of a model for me. In Switzerland, exactly three university hospitals are authorized to carry out allogeneic stem cell transplants and no one else. And this has been regulated by law. This has some very big, smart advantages: The result, the quality that is achieved, is very good. The clinics are not constantly poaching specialists from one another. This is a huge problem in the field of stem cell transplantation because there is only a very limited number of suitable specialists. And there is a very large fluctuation in the sector. For example, the doctor who transplanted me at the University Hospital in x was then temporarily based in y. She is now based in z. This means that there is an incredible amount of movement, which certainly has consequences for the medical profession, but also for the quality of the work. Because you simply need continuity and experience. And that's what's missing there.”*	**Employee fluctuation**
**Medical society**	"So, I see a pancreatic carcinoma every week, let me give you an example, which is rejected as inoperable elsewhere. It goes into the palliative track, life expectancy six months. And if you do this more often and have more expertise, you can operate on these patients very frequently. And that is of course a problem for everyone, but especially for surgeons. We all have a huge ego. You don't say: "I can't do this", you say: "You can't do that". And if you centralize, like in other countries, you wouldn't have this problem."	**Access to treatment/ therapy options (number, quality)**
**Medical society**	"And then we are back to the three challenges we have. We need units that have sufficient staff to fulfil working time laws and collective bargaining conditions and also the demands of the staff. At the same time, they have so many, I'll stick to births now, or pregnant women, but let's stick to births, it's easier to have routines that enable rare complications to be managed successfully. And it's not enough to have a birth every other day. The rare complications are, if they occur one in 500 or one in 700 times, then they will only occur every two years. And then you can't expect the necessary chains of management decisions to be made automatically in the same way as in a clinic that has them every week or every month. And that is a definition, so to speak, of what we want to achieve. We need units that are large enough and employ sufficient staff to keep them happy.“	**Staffing levels, Complication management (quality), Employee job satisfaction**

**Statutory health insurance fund**	"One disadvantage is perhaps, on the one hand, the perceived disadvantage that people have when everything is centralized and especially in regions, as Mr x has already said, where there are gradually no more GPs or specialists in the outpatient sector and so on. So that's perhaps the feeling you get: Oh God, and now the small clinic is going to go. Simply the feeling of insecurity: Oh, I no longer have a provider who could help me if the worst comes to the worst."	**Psychological safety of the population with respect to healthcare**
