# Supplementary material for: Development of a core outcome set for studies on centralization of healthcare services
Source: BMC Health Serv Res. 2026 Jun 9;26:810. doi: 10.1186/s12913-026-14861-z (PMC13255221; doi:10.1186/s12913-026-14861-z)
Supplement: Supplementary file 7 — Supplementary Material 7 [file 12913_2026_14861_MOESM7_ESM.pdf]

Online survey “Centralization of inpatient healthcare services” (own translation)
Health services researchers

Bundesministerium
für Bildung
und Forschung

ZVF-BB
Zentrum für Versorgungsforschung Brandenburg

(FKZ 01KG2107)

Dear colleagues from the field of health services research,

Welcome to our survey, with which we would like to take a further step on the way to developing a Core Outcome Set (COS) for studies on the centralization of inpatient healthcare services. **Accordingly, the central aim of this survey is to collect important outcomes in studies on the centralization of inpatient healthcare services.** We look forward to your participation and your expertise. Further information on the project “MIVOS – The effects of minimum volume standards in hospitals” can be found, for example, in the database of the COMET Initiative at https://www.comet-initiative.org/Studies/Details/2077 or at this link

Link to leaflet of the project

Below are some general and organizational notes on this survey: It takes about 5-10 minutes to complete all the questions. On the last page of this survey, you also have the opportunity to leave us your comments and feedback. We will also ask you to leave your email address for the subsequent Delphi survey for developing the final COS. However, participation in the Delphi survey is not a prerequisite for participation in this study.

Thank you for supporting our project!

Stefanie Pfisterer-Heise, Julia Scharfe and Prof. Dr. Dawid Pieper

---

THIS IS AN ANONYMOUS SURVEY.

No personal information about you will be saved in the survey responses unless explicitly asked for in a question. If you have used an access code for this survey, you can be sure that the access code has not been stored together with the data. It is kept in a separate table and is only updated to record whether or not you have completed this survey. There is no way to merge the access codes with the survey results.

DATA PROTECTION DECLARATION
I hereby consent to my participation in the survey.

Your participation is anonymous and voluntary. Non-participation will not affect you negatively in any way. The completed questionnaires are only accessible to employees of

Brandenburg Medical School Theodor Fontane and the results will only be published in aggregated form. By completing and submitting this questionnaire, we explicitly assure you that your data will be fully protected. Neither email addresses (unless you explicitly state so at the end of this survey) nor IP addresses will be stored. The data is subject to statutory regulations, such as the Federal Data Protection Act (BDSG) and the General Data Protection Regulation (DSVGO, GDPR). In order to ensure anonymity, we ask you to refrain from providing personal or personally identifiable data in the free text fields.

To open the survey, please accept our privacy policy.

Checkbox

This survey contains 14 questions.

Outcomes in studies on the centralization of inpatient healthcare services

Various outcomes are currently measured and reported in studies on the centralization of inpatient healthcare services. These include:

Outcomes that directly affect patients, e.g.

- Mortality
- Health-related quality of life with its somatic, psychological and social dimensions
- Patient compliance

Outcomes that affect access to treatment and the treatment itself, e.g.

- Travel time from the patient’s place of residence to the hospital
- Length of stay
- Guideline adherence

Outcomes that affect service providers, e.g.

- Amount of work involved
- Physical and mental strain on employees

Outcomes that affect the healthcare system, e.g.

- Continuity of care (e.g. by making it more difficult to reach aftercare centers and service providers due to greater distances)
- Coordination of care (e.g. existing co-operations between hospitals and other service providers could change)
- Quality of emergency care (e.g. emergency care could be jeopardized by greater centralization).

In your opinion, which (further) outcomes should be measured and reported in studies on the centralization of inpatient healthcare services? Please consider all interest groups and all areas of the healthcare system.

Free text field

Information on your thematic background

Have you already conducted research on the *centralization of inpatient healthcare services*?

Yes
No

How would you rate your expertise on the subject of *centralization of inpatient healthcare services*?

1= no expertise
2= little expertise
3= some expertise
4= high expertise
5= very high expertise

Have you already conducted research on *minimum volume standards*?

Yes
No

How would you rate your expertise on the subject of *minimum volume standards*?

1= no expertise
2= little expertise
3= some expertise
4= high expertise
5= very high expertise

Do you work in the clinical care of patients?

Yes

No

Socio-demographic information

How old are you?

Only numbers may be entered in this field.
Free text field

What is your gender?

Please select only one of the following answers:

Male
Female
Diverse
Not specified

How long have you been working in health services research?

Please select only one of the following answers:

Less than 1 year to 3 years
Over 3 to 5 years
Over 5 to 10 years
Over 10 years

What is your current professional position?

Please select only one of the following answers:

PhD candidate
Research assistant without a PhD
Post-doc
Professor

Which subject did you study (Diploma, B.Sc., M.Sc.)?

Please select all applicable answers:

Medicine
Psychology
Sociology

Public Health
Other: *Free text field*

Do you have any comments on this study? If so, please let us know here.

Free text field

May we contact you again for the invitation to the Delphi study?

Yes
No

Please contact me at the following email address.

Free text field

You have reached the end of our survey. Thank you very much for your participation and good luck with your research.
