# Supplementary material for: Development of a core outcome set for studies on centralization of healthcare services
Source: BMC Health Serv Res. 2026 Jun 9;26:810. doi: 10.1186/s12913-026-14861-z (PMC13255221; doi:10.1186/s12913-026-14861-z)
Supplement: Supplementary file 8 — Supplementary Material 8 [file 12913_2026_14861_MOESM8_ESM.pdf]

Delphi survey I “Centralization of inpatient healthcare services” (own translation)
All interest holder groups

Bundesministerium für Bildung und Forschung

MHB ZVF-BB Zentrum für Versorgungsforschung Brandenburg

(FKZ 01KG2107)

Dear patient representatives, representatives of the medical societies, representatives of the statutory health insurance funds and health services researchers,

Welcome to our first Delphi survey, a structured process aimed at reaching consensus among different experts. As you know, the aim of our project is to develop **a core outcome set for studies on the centralization of inpatient healthcare services**. In other words, we want to reach consensus on which outcomes/ parameters should be measured and reported in studies on centralization. In the following, we present a list of outcomes/ parameters that can be influenced by the centralization of inpatient healthcare services. All of the listed outcomes were obtained with your help in group discussions, interviews and an online survey.

Today we would like to ask you to rate these outcomes in terms of their importance in studies on the centralization of inpatient healthcare services. It does not matter whether data are available or not. Please make your judgement based solely on the importance of the outcome. Please also note that throughout the project we are adopting an international perspective. In a second Delphi survey (probably in September), we will then show you the summarized, anonymous results of this first survey and ask you once again to rate the outcomes in terms of their importance in order to reach consensus for the core outcome set.

Below are some general and organizational notes on this survey: The survey takes about 25 minutes to complete. If you wish to make changes to your ratings, you have the option of navigating within the survey (not via the browser) using the “back” and “next” buttons. On the last page of this survey, you also have the opportunity to leave us your comments on the survey.

Thank you for continuing to support our project with your time and expertise.

Stefanie Pfisterer-Heise, Julia Scharfe, Alexander Pachanov and Prof. Dr. Dawid Pieper

This survey contains 54 questions.

Delphi survey I “Centralization of inpatient healthcare services” (own translation)
All interest holder groups

Which of the following interest holder groups that we surveyed as part of this project do you belong to? Answering this question is very important, as we would like to show you the average values of each group for each outcome in the second Delphi survey (probably in September). To do this, we need to know which of the groups you belong to.

Note: If you can assign yourself to more than one group (e.g. physicians and health services researchers), please assign yourself to the group for which you participated in this project. Thank you very much.

Please select only one of the following answers:

Patient representatives

Representatives of statutory health insurance funds

Representatives of medical societies

Health services researchers

In the following, we present a total of 48 outcomes in ten randomly ordered categories, such as patient-related outcomes, outcomes related to access to healthcare, etc.

Please rate these outcomes in terms of their importance in studies on the centralization of inpatient healthcare services. In other words: Imagine you were conducting or reading a study on the centralization of inpatient healthcare services. How important would it be to you that the following outcomes were measured and reported?

Please rate all the outcomes presented on a scale of

1-3= Not important
4-6= Important but not critical
7-9= Critically important

To get the most accurate picture of your assessment, please use the nine levels of the scale fully.

In addition to your ratings, you will find a comment field for each outcome where you have the opportunity to state your reasons for your rating. In the second Delphi survey, we will then show you the aggregated values and the anonymous reasons from this first Delphi round and ask you once again to rate the outcomes in terms of their importance.

The outcome sets for the individual interest holder groups will then be made up of outcomes that 75 per cent or more participants in an interest holder group have rated as “Critically important” (values 7 to 9) (minimum 5 participants per group); the final core outcome set will be made up of outcomes that are included in at least two outcome sets of the interest

holder groups. Further information on the development of the core outcome set can be found at

Link to the study protocol

In the field below you have the opportunity to leave us a comment, for example regarding the comprehensibility of the instructions.

Please enter your answer her:

Free text field

Patient related outcomes

Quality of life
Please select only one of the following answers:

1= Not important
2
3
4
5
6
7
8
9= Critically important

Please write a comment on your rating
Free text field

Morbidity (complications, health complaints)
Please select only one of the following answers:

1= Not important
2
3
4
5
6
7
8
9= Critically important

Please write a comment on your rating
Free text field

Mortality
Please select only one of the following answers:

1= Not important
2
3
4
5
6
7
8
9= Critically important

Please write a comment on your rating
Free text field

Satisfaction with healthcare
Please select only one of the following answers:

1= Not important
2
3
4
5
6
7
8
9= Critically important

Please write a comment on your rating
Free text field

Outcomes related to the quality of care

Adherence to guidelines, clinical pathways of concepts
Please select only one of the following answers:

1= Not important
2

3
4
5
6
7
8
9= Critically important

Please write a comment on your rating
Free text field

Outpatient care (quality)
Please select only one of the following answers:

1= Not important
2
3
4
5
6
7
8
9= Critically important

Please write a comment on your rating
Free text field

Relationship between patient and members of the treatment team (quality)
Please select only one of the following answers:

1= Not important
2
3
4
5
6
7
8
9= Critically important

Please write a comment on your rating
Free text field

Diagnostics (quality)

Delphi survey I “Centralization of inpatient healthcare services” (own translation)
All interest holder groups

Please select only one of the following answers:

1= Not important
2
3
4
5
6
7
8
9= Critically important

Please write a comment on your rating
Free text field

Indication (quality)
Please select only one of the following answers:

1= Not important
2
3
4
5
6
7
8
9= Critically important

Please write a comment on your rating
Free text field

Complication management (quality)
Please select only one of the following answers:

1= Not important
2
3
4
5
6
7
8
9= Critically important

Please write a comment on your rating

Free text field

Multi-professional care/ Multi-disciplinarity of care
Please select only one of the following answers:

1= Not important
2
3
4
5
6
7
8
9= Critically important

Please write a comment on your rating
Free text field

Emergency care (quality)
Please select only one of the following answers:

1= Not important
2
3
4
5
6
7
8
9= Critically important

Please write a comment on your rating
Free text field

Degree of structuring of care pathways
Please select only one of the following answers:

1= Not important
2
3
4
5

6
7
8
9= Critically important

Please write a comment on your rating
Free text field

Therapy success/ treatment quality
Please select only one of the following answers:

1= Not important
2
3
4
5
6
7
8
9= Critically important

Please write a comment on your rating
Free text field

Outcomes related to the utilization of health services

Visits to outpatient physicians (number)
Please select only one of the following answers:

1= Not important
2
3
4
5
6
7
8
9= Critically important

Please write a comment on your rating
Free text field

Surgical procedures (number)
Please select only one of the following answers:

1= Not important
2
3
4
5
6
7
8
9= Critically important

Please write a comment on your rating
Free text field

Inpatient treatments (number, duration)
Please select only one of the following answers:

1= Not important
2
3
4
5
6
7
8
9= Critically important

Please write a comment on your rating
Free text field

Outcomes related to access to healthcare

Waiting time
Please select only one of the following answers:

1= Not important
2
3
4
5
6
7
8
9= Critically important

Please write a comment on your rating
Free text field

Digitalization/ telemedicine
Please select only one of the following answers:

1= Not important
2
3
4
5
6
7
8
9= Critically important

Please write a comment on your rating
Free text field

Proximity of care to place of residence (distance, travel time)
Please select only one of the following answers:

1= Not important
2
3
4
5
6
7
8
9= Critically important

Please write a comment on your rating
Free text field

Treatment/ therapy options (number
Please select only one of the following answers:

1= Not important
2
3
4
5
6

7
8
9= Critically important

Please write a comment on your rating
Free text field

Outcomes related to the use of health care resources

Outpatient/ regional providers (number)
Please select only one of the following answers:

1= Not important
2
3
4
5
6
7
8
9= Critically important

Please write a comment on your rating
Free text field

Hospital beds (number)
Please select only one of the following answers:

1= Not important
2
3
4
5
6
7
8
9= Critically important

Please write a comment on your rating
Free text field

Costs for the healthcare system
Please select only one of the following answers:

1= Not important
2
3
4
5
6
7
8
9= Critically important

Please write a comment on your rating
Free text field

Cost efficiency of the healthcare system
Please select only one of the following answers:

1= Not important
2
3
4
5
6
7
8
9= Critically important

Please write a comment on your rating
Free text field

Hospitals (number)
Please select only one of the following answers:

1= Not important
2
3
4
5
6
7
8
9= Critically important

Please write a comment on your rating
Free text field

Staffing levels
Please select only one of the following answers:

1= Not important
2
3
4
5
6
7
8
9= Critically important

Please write a comment on your rating
Free text field

Technical equipment of the inpatient providers
Please select only one of the following answers:

1= Not important
2
3
4
5
6
7
8
9= Critically important

Please write a comment on your rating
Free text field

Outcomes related to the use of non-health care resources

Visitors for patients
Please select only one of the following answers:

1= Not important
2
3
4

5
6
7
8
9= Critically important

Please write a comment on your rating
Free text field

Accommodation options for family/ relatives close to hospital
Please select only one of the following answers:

1= Not important
2
3
4
5
6
7
8
9= Critically important

Please write a comment on your rating
Free text field

Health care provider outcomes

Employee workload
Please select only one of the following answers:

1= Not important
2
3
4
5
6
7
8
9= Critically important

Please write a comment on your rating
Free text field

Employee turnover
Please select only one of the following answers:

1= Not important
2
3
4
5
6
7
8
9= Critically important

Please write a comment on your rating
Free text field

Employee job satisfaction
Please select only one of the following answers:

1= Not important
2
3
4
5
6
7
8
9= Critically important

Please write a comment on your rating
Free text field

Training positions for junior physicians (quality, number)
Please select only one of the following answers:

1= Not important
2
3
4
5
6
7
8
9= Critically important

Please write a comment on your rating
Free text field

Routine of members of the treatment team (number of cases)
Please select only one of the following answers:

1= Not important
2
3
4
5
6
7
8
9= Critically important

Please write a comment on your rating
Free text field

Routine of surgeons (number of cases)
Please select only one of the following answers:

1= Not important
2
3
4
5
6
7
8
9= Critically important

Please write a comment on your rating
Free text field

Outcomes related to equity of healthcare

Health care equity independent of the region
Please select only one of the following answers:

1= Not important
2
3
4
5
6
7
8
9= Critically important

Please write a comment on your rating
Free text field

Health care equity independent of patients’ sociodemographic factors
Please select only one of the following answers:

1= Not important
2
3
4
5
6
7
8
9= Critically important

Please write a comment on your rating
Free text field

Adverse effects of harms

Revisions
Please select only one of the following answers:

1= Not important
2
3
4
5
6
7
8

9= Critically important

Please write a comment on your rating
Free text field

Adverse events
Please select only one of the following answers:

1= Not important
2
3
4
5
6
7
8
9= Critically important

Please write a comment on your rating
Free text field

Readmissions
Please select only one of the following answers:

1= Not important
2
3
4
5
6
7
8
9= Critically important

Please write a comment on your rating
Free text field

Wound infections
Please select only one of the following answers:

1= Not important

2
3
4
5
6
7
8
9= Critically important

Please write a comment on your rating
Free text field

Diverse outcomes

Shifts in the provision of hospital services
Please select only one of the following answers:

1= Not important
2
3
4
5
6
7
8
9= Critically important

Please write a comment on your rating
Free text field

Co-operations between health service providers
Please select only one of the following answers:

1= Not important
2
3
4
5
6
7
8
9= Critically important

Please write a comment on your rating
Free text field

Psychological safety of the population with respect to healthcare
Please select only one of the following answers:

1= Not important
2
3
4
5
6
7
8
9= Critically important

Please write a comment on your rating
Free text field

Outpatient care physicians' knowledge about healthcare
Please select only one of the following answers:

1= Not important
2
3
4
5
6
7
8
9= Critically important

Please write a comment on your rating
Free text field

Patients' knowledge about healthcare
Please select only one of the following answers:

1= Not important
2
3
4

5
6
7
8
9= Critically important

Please write a comment on your rating
Free text field

Transparency (e.g. of treatment quality and clinical pathways)
Please select only one of the following answers:

1= Not important
2
3
4
5
6
7
8
9= Critically important

Please write a comment on your rating
Free text field

Are there any other outcomes not mentioned in this list that you think should be measured and reported in studies on the centralization of inpatient healthcare services?

If so, please enter your answer here:

Free text field

Socio-demographic information

How old are you?

Only numbers may be entered in this field, please enter your answer here:

What is your gender?

Please select only one of the following answers:

Male
Female
Diverse

Here you have the opportunity to leave us your comment.

Please enter your answer here:

Thank you for your participation in our first Delphi survey. We would be very pleased if you would also take part in the second Delphi survey in September so that the results obtained are based on a secure methodological foundation. We will then send you the corresponding link again by e-mail and wish you a good time until then! If you have any questions in the meantime, please do not hesitate to contact us at stefanie.heise@mhb-fontane.de
