# Supplementary material for: Development of a core outcome set for studies on centralization of healthcare services
Source: BMC Health Serv Res. 2026 Jun 9;26:810. doi: 10.1186/s12913-026-14861-z (PMC13255221; doi:10.1186/s12913-026-14861-z)
Supplement: Supplementary file 9 — Supplementary Material 9 [file 12913_2026_14861_MOESM9_ESM.pdf]

Delphi I & Delphi 2 Outcome Mean Values and Outcome Inclusion in COS

Delphi II/ Delphi I*	Delphi I<br><br>M_{total} (SD_{total})	Delphi I<br><br>M_{group} (SD_{group})	Delphi I<br><br>Outcome "Critically important" (%)	Delphi I<br><br>Outcome included	Delphi II<br><br>M_{total} (SD_{total})	Delphi II<br><br>M_{group} (SD_{group})	Delphi II<br><br>Outcome "Critically important" (%)	Delphi II<br><br>Outcome included	COS<br><br>Outcome included
**Patient-related outcomes**									
Health-related quality of life/<br>Quality of life*	7.89 (0.48)	*HI*: 7.38 (1.60)<br>*MS*: 7.83 (0.98)<br>*PO*: 8.67 (0.49)<br>*HR*: 7.70 (1.06)	*HI:* 63<br>*MS:* 100<br>*PO:* 100<br>*HR:* 90	Yes	7.96 (0.35)	*HI*: 7.78 (0.97)<br>*MS*: 7.71 (0.49)<br>*PO*: 8.56 (1.01)<br>*HR*: 7.78 (1.48)	*HI*: 89<br>*MS*: 100<br>*PO*: 89<br>*HR*: 78	Yes	Yes
Morbidity (complications, health complaints)	8.12 (0.26)	*HI*: 8.00 (1.20)<br>*MS*: 8.17 (0.98)<br>*PO*: 8.50 (0.90)<br>*HR*: 7.80 (1.40)	*HI:* 88<br>*MS:* 100<br>*PO:* 92<br>*HR:* 80	Yes	8.03 (0.16)	*HI*: 8.11 (0.33)<br>*MS*: 8.00 (0.82)<br>*PO*: 8.22 (0.97)<br>*HR*: 7.78 (0.97)	*HI*: 100<br>*MS*: 100<br>*PO*: 89<br>*HR*: 89	Yes	Yes
Mortality	8.12 (0.37)	*HI*: 8.63 (1.06)<br>*MS*: 7.83 (1.47)	*HI:* 88<br>*MS:* 67<br>*PO:* 92<br>*HR:* 90	Yes	7.96 (0.51)	*HI*: 8.00 (0.87)<br>*MS*: 7.57 (0.98)	*HI*: 100<br>*MS*: 86<br>*PO*: 100<br>*HR*: 88	Yes	Yes

Delphi II/ Delphi I*	Delphi I<br>*M*$_{total}$ (*SD*$_{total}$)	Delphi I<br>*M*$_{group}$ (*SD*$_{group}$)	Delphi I<br>Outcome "Critically important" (%)	Delphi I<br>Outcome included	Delphi II<br>*M*$_{total}$ (*SD*$_{total}$)	Delphi II<br>*M*$_{group}$ (*SD*$_{group}$)	Delphi II<br>Outcome "Critically important" (%)	Delphi II<br>Outcome included	COS<br>Outcome included
		PO: 8.33 (0.98)<br>*HR*: 7.70 (1.49)				*PO*: 8.78 (0.44)<br>*HR*: 7.50 (1.60)			
Satisfaction with healthcare	6.60 (0.39)	*HI*: 6.00 (2.07)<br>*MS*: 6.50 (1.87)<br>*PO*: 7.00 (1.28)<br>*HR*: 6.90 (1.73)	*HI:* 50<br>*MS:* 50<br>*PO:* 75<br>*HR:* 50	No	6.46 (0.22)	*HI*: 6.22 (1.48)<br>*MS*: 6.29 (0.49)<br>*PO*: 6.78 (1.20)<br>*HR*: 6.56 (0.73)	*HI*: 44<br>*MS*: 29<br>*PO*: 56<br>*HR*: 44	No	No
**Outcomes related to the quality of care**									
Adherence to guidelines, clinical pathways or concepts	7.61 (0.42)	*HI:* 8.25 (0.71)<br>*MS:* 7.33 (1.03)<br>*PO:* 7.17 (1.19)<br>*HR:* 7.70 (0.95)	*HI:* 100<br>*MS:* 83<br>*PO:* 58<br>*HR:* 80	Yes	7.73 (0.09)	*HI*: 7.78 (0.67)<br>*MS*: 7.57 (0.98)<br>*PO*: 7.78 (0.67)<br>*HR*: 7.78 (0.97)	HI: 100<br>MS: 86<br>PO: 100<br>HR: 89	Yes	Yes
Outpatient care in ambulatory settings	7.15 (0.37)	*HI:* 6.88 (1.46)	*HI:* 63<br>*MS:* 67<br>*PO:* 75	No	7.24 (0.21)	*HI*: 7.33 (1.00)	HI: 89<br>MS: 100<br>PO: 78	Yes	Yes

**Delphi II/ Delphi I***	**Delphi I**<br><br>***M*** M_{total} (SD_{total})	**Delphi I**<br><br>M_{group} (SD_{group})	**Delphi I**<br><br>**Outcome "Critically important" (%)**	**Delphi I**<br><br>**Outcome included**	**Delphi II**<br><br>M_{total} (SD_{total})	**Delphi II**<br><br>M_{group} (SD_{group})	**Delphi II**<br><br>**Outcome "Critically important" (%)**	**Delphi II**<br><br>**Outcome included**	**COS**<br><br>**Outcome included**
(quality)/ Outpatient care (quality)*		*MS:* 7.17 (1.17)<br>*PO:* 7.75 (1.42)<br>*HR:* 6.80 (1.48)	*HR:* 50			*MS*: 7.29 (0.76)<br>*PO*: 7.44 (1.01)<br>*HR*: 6.89 (1.17)	HR: 67		
Relationship between patient and members of the treatment team (quality)	6.51 (0.58)	*HI*: 5.75 (2.19)<br>*MS:* 6.67 (1.97)<br>*PO:* 7.33 (1.92)<br>*HR:* 6.30 (1.34)	*HI:* 38<br>*MS:* 50<br>*PO:* 75<br>*HR:* 60	No	6.48 (0.49)	*HI*: 6.22 (1.30)<br>*MS*: 6.57 (0.98)<br>*PO*: 7.22 (0.97)<br>*HR*: 5.89 (1.05)	HI: 33<br>MS: 57<br>PO: 78<br>HR: 33	No	No
Diagnostics (quality)	7.87 (0.51)	*HI:* 8.25 (0.89)<br>*MS*: 7.33 (1.21)<br>*PO*: 8.50 (1.00)<br>HR: 7.40 (1.26)	*HI:* 100<br>*MS:* 67<br>*PO:* 92<br>*HR:* 90	Yes	8.08 (0.21)	*HI*: 7.89 (0.60)<br>*MS*: 8.00 (0.58)<br>*PO*: 8.44 (0.73)<br>*HR*: 8.00 (1.00)	HI: 100<br>MS: 100<br>PO: 100<br>HR: 89	Yes	Yes
Indication (quality)	7.97 (0.28)	HI: 8.25 (0.89)	HI: 100<br>MS: 83	Yes	8.17 (0.18)	*HI*: 8.33 (0.71)	HI: 100<br>MS: 100	Yes	Yes

**Delphi II/ Delphi I***	**Delphi I**<br><br>***M_{total}* (*SD_{total}*)**	**Delphi I**<br><br>***M_{group}* (*SD_{group}*)**	**Delphi I**<br><br>**Outcome "Critically important" (%)**	**Delphi I**<br><br>**Outcome included**	**Delphi II**<br><br>***M_{total}* (*SD_{total}*)**	**Delphi II**<br><br>***M_{group}* (*SD_{group}*)**	**Delphi II**<br><br>**Outcome "Critically important" (%)**	**Delphi II**<br><br>**Outcome included**	**COS**<br><br>**Outcome included**
		MS: 7.67 (1.51)<br>PO: 8.25 (0.97)<br>HR: 7.70 (1.25)	PO: 92<br>HR: 90			*MS*: 8.14 (0.69)<br>*PO*: 8.33 (0.50)<br>*HR*: 7.89 (1.05)	PO: 100<br>HR: 89		
Complication management (quality)	7.88 (0.30)	HI: 7.88 (1.13)<br>MS: 7.50 (1.52)<br>PO: 8.33 (0.78)<br>HR: 7.80 (1.32)	HI: 88<br>MS: 83<br>PO: 100<br>HR: 70	Yes	7.99 (0.26)	*HI*: 7.78 (1.09)<br>*MS*: 7.86 (0.69)<br>*PO*: 8.44 (0.53)<br>*HR*: 7.89 (1.05)	HI: 78<br>MS: 100<br>PO: 100<br>HR: 89	Yes	Yes
Multi-professional care/ multi-disciplinarity of care	7.55 (0.11)	HI: 7.63 (0.92)<br>MS: 7.50 (1.76)<br>PO: 7.67 (1.72)<br>HR: 7.40 (1.17)	HI: 100<br>MS: 67<br>PO: 67<br>HR: 80	Yes	7.54 (0.38)	*HI*: 7.67 (1.00)<br>*MS*: 7.71 (0.49)<br>*PO*: 7.89 (1.05)<br>*HR*: 6.89 (1.54)	HI: 89<br>MS: 100<br>PO: 89<br>HR: 67	Yes	Yes
Emergency care (quality)	7.74 (0.60)	HI: 7.63 (1.41)	HI: 75<br>MS: 83	Yes	8.07 (0.59)	*HI*: 7.44 (1.13)	HI: 89<br>MS: 100	Yes	Yes

**Delphi II/ Delphi I***	**Delphi I**<br><br>***M**total*<br>**(*SD*total)**	**Delphi I**<br><br>***M*group**<br>***(SDgroup)***	**Delphi I**<br><br>**Outcome "Critically important" (%)**	**Delphi I**<br><br>**Outcome included**	**Delphi II**<br><br>***M**total*<br>**(*SD*total)**	**Delphi II**<br><br>***M*group**<br>***(SDgroup)***	**Delphi II**<br><br>**Outcome "Critically important" (%)**	**Delphi II**<br><br>**Outcome included**	**COS**<br><br>**Outcome included**
		MS: 7.50 (1.05)<br>PO: 8.73 (0.65)<br>HR: 7.10 (1.52)	PO: 100<br>HR: 60			*MS*: 7.71 (0.76)<br>*PO*: 9.00 (0.00)<br>*HR*: 8.13 (0.99)	PO: 100<br>HR: 88		
Clinical pathways (quality)/ Degree of structuring of care pathways*	6.54 (0.53)	HI: 6.75 (1.49)<br>MS: 6.67 (1.37)<br>PO: 7.09 (1.81)<br>HR: 5.67 (1.41)	HI: 50<br>MS: 50<br>PO: 73<br>HR: 33	No	7.14 (0.14)	*HI*: 7.00 (0.87)<br>*MS*: 7.00 (0.82)<br>*PO*: 7.22 (1.20)<br>*HR*: 7.33 (1.00)	HI: 67<br>MS: 71<br>PO: 78<br>HR: 78	Yes	Yes
Individual treatment success/ Therapy success, treatment quality*	8.26 (0.13)	HI: 8.38 (0.74)<br>MS: 8.17 (0.98)<br>PO: 8.42 (0.79)<br>HR: 8.10 (0.99)	HI: 100<br>MS: 100<br>PO: 100<br>HR: 100	Yes	8.06 (0.13)	*HI*: 7.89 (0.93)<br>*MS*: 8.14 (0.69)<br>*PO*: 8.22 (0.67)<br>*HR*: 8.00 (0.87)	HI: 89<br>MS: 100<br>PO: 100<br>HR: 89	Yes	Yes
Adverse effects (revisions, readmissions,	7.55 (0.17)	HI: 7.38 (1.06)	HI: 88<br>MS: 83	Yes	7.68 (0.26)	*HI*: 7.67 (0.87)	HI: 89<br>MS: 86	Yes	Yes

Delphi II/ Delphi I*	Delphi I<br><br>***M****total*<br>**(*SD*total)**	Delphi I<br><br>***M*group**<br>***(SDgroup)***	Delphi I<br><br>**Outcome "Critically important" (%)**	Delphi I<br><br>**Outcome included**	Delphi II<br><br>***M****total*<br>**(*SD*total)**	Delphi II<br><br>***M*group**<br>***(SDgroup)***	Delphi II<br><br>**Outcome "Critically important" (%)**	Delphi II<br><br>**Outcome included**	COS<br><br>**Outcome included**
wound infections)/<br>Adverse effects*		MS: 7.83 (1.17)<br>PO: 7.50 (1.51)<br>HR: 7.50 (1.84)	PO: 80<br>HR: 90			*MS*: 7.29 (0.76)<br>*PO*: 7.78 (0.44)<br>*HR*: 8.00 (1.00)	PO: 100<br>HR: 89		
Revisions (only)*	*7.41 (0.59)*	*HI: 8.13 (0.83)*<br>*MS*: *6.67 (1.37)*<br>*PO*: *7.83 (1.34)*<br>*HR*: *7.00 (1.66)*	*HI: 100*<br>*MS: 67*<br>*PO: 83*<br>*HR: 78*	Yes			*HI:*<br>*MS:*<br>*PO:*<br>*HR:*		
*Readmissions (only *)*	*6.85 (0.68)*	*HI*: *7.75 (1.04)*<br>*MS*: *5.83 (0.98)*<br>*PO*: *6.91 (1.45)*<br>*HR*: *6.90 (1.60)*	*HI: 88*<br>*MS: 33*<br>*PO: 55*<br>*HR: 50*	No			*HI:*<br>*MS:*<br>*PO:*<br>*HR:*		
Wound infections (only)*	*7.45 (0.56)*	*HI*: *7.88 (1.13)*	*HI: 88*<br>*MS: 83*	Yes			*HI:*<br>*MS:*		

Delphi II/ Delphi I*	Delphi I<br><br>***M****total*<br>(***SD***total)	Delphi I<br><br>***M***group<br>***(SD****group)*	Delphi I<br><br>Outcome "Critically important" (%)	Delphi I<br><br>Outcome included	Delphi II<br><br>***M****total*<br>(***SD***total)	Delphi II<br><br>***M***group<br>***(SD****group)*	Delphi II<br><br>Outcome "Critically important" (%)	Delphi II<br><br>Outcome included	COS<br><br>Outcome included
		MS: *7.83 (0.98)*<br>*PO*: *7.58 (1.00)*<br>*HR*: *6.50 (2.12)*	*PO: 92*<br>*HR: 60*				*PO:*<br>*HR:*		
**Outcomes related to the utilization of health services**									
Visits to outpatient physicians (number)	5.59 (0.95)	HI: 5.75 (1.39)<br>MS: 4.00 (1.22)<br>PO: 6.42 (1.38)<br>HR: 6.20 (1.55)	HI: 25<br>MS: 0<br>PO: 42<br>HR: 40	No	5.96 (0.48)	*HI*: 5.67 (1.41)<br>*MS*: 5.33 (1.75)<br>*PO*: 6.33 (1.22)<br>*HR*: 6.50 (1.60)	*HI*: 22<br>*MS*: 33<br>*PO*: 44<br>HR: 50	No	No
Surgical procedures (number)	6.89 (0.68)	HI: 7.00 (1.07)<br>MS: 5.80 (1.92)<br>PO: 7.67 (1.23)<br>HR: 7.10 (1.37)	HI: 63<br>MS: 40<br>PO: 75<br>HR: 80	Yes	7.37 (0.43)	*HI*: 7.11 (0.93)<br>*MS*: 7.50 (1.05)<br>*PO*: 8.00 (1.00)<br>*HR*: 6.88 (1.46)	HI: 89<br>MS: 83<br>PO: 89<br>HR: 75	Yes	Yes

**Delphi II/ Delphi I***	**Delphi I**<br><br>***M*** M_{total} (SD_{total})	**Delphi I**<br><br>M_{group} (SD_{group})	**Delphi I**<br><br>**Outcome “Critically important” (%)**	**Delphi I**<br><br>**Outcome included**	**Delphi II**<br><br>M_{total} (SD_{total})	**Delphi II**<br><br>M_{group} (SD_{group})	**Delphi II**<br><br>**Outcome “Critically important” (%)**	**Delphi II**<br><br>**Outcome included**	**COS**<br><br>**Outcome included**
Inpatient treatments (number, duration)	6.11 (0.85)	HI: 6.38 (1.77)<br>MS: 4.80 (0.84)<br>PO: 7.17 (1.34)<br>HR: 6.10 (0.74)	HI: 38<br>MS: 0<br>PO: 67<br>HR: 30	No	6.52 (0.54)	*HI*: 6.00 (1.00)<br>*MS*: 6.00 (1.67)<br>*PO*: 7.22 (1.39)<br>*HR*: 6.88 (1.55)	*HI*: 22<br>*MS*: 33<br>*PO*: 67<br>*HR*: 63	No	No
**Outcomes related to access and equity of health care/ Outcomes related to access of health care***									
Waiting time from indication to treatment, surgical procedure/ Waiting time*	6.29 (0.85)	HI: 5.13 (1.13)<br>MS: 5.83 (2.32)<br>PO: 7.00 (1.54)<br>HR: 7.20 (0.63)	HI: 13<br>MS: 33<br>PO: 67<br>HR: 90	No	6.88 (0.59)	*HI*: 6.22 (0.97)<br>*MS*: 6.43 (1.27)<br>*PO*: 7.67 (0.87)<br>*HR*: 7.22 (1.20)	HI: 33<br>MS: 43<br>PO: 89<br>HR: 67	No	No
Digitilization/ telemedicine	6.49 (0.20)	*HI*: 6.63 (1.77)<br>*MS*: 6.17 (1.17)<br>*PO*: 6.67 (1.78)	HI: 63<br>MS: 50<br>PO: 58<br>HR: 50	No	6.77 (0.48)	*HI*: 6.67 (1.58)<br>*MS*: 6.29 (0.49)<br>*PO*: 7.56 (1.24)	HI: 78<br>MS: 29<br>PO: 67<br>HR: 44	No	No

**Delphi II/ Delphi I***	**Delphi I**<br><br>***M**total*<br>**(*SD*total)**	**Delphi I**<br><br>***M*group**<br>***(SDgroup)***	**Delphi I**<br><br>**Outcome "Critically important" (%)**	**Delphi I**<br><br>**Outcome included**	**Delphi II**<br><br>***M**total*<br>**(*SD*total)**	**Delphi II**<br><br>***M*group**<br>***(SDgroup)***	**Delphi II**<br><br>**Outcome "Critically important" (%)**	**Delphi II**<br><br>**Outcome included**	**COS**<br><br>**Outcome included**
		HR: 6.50 (1.90)				*HR*: 6.56 (1.59)			
Proximity of care to place of residence (distance, travel time)	5.04 (0.58)	*HI*: 4.75 (1.16)<br>*MS*: 4.33 (1.21)<br>*PO*: 5.17 (1.85)<br>*HR*: 5.90 (1.91)	HI: 0<br>MS: 0<br>PO: 17<br>HR: 50	No	4.78 (0.43)	*HI*: 4.33 (1.12)<br>*MS*: 4.43 (0.98)<br>*PO*: 5.00 (1.12)<br>*HR*: 4.89 (2.20)	HI: 0<br>MS: 0<br>PO: 11<br>HR: 25	No	No
Access to treatment, therapy options (number, quality)/ Treatment, therapy options (number, quality)*	7.27 (0.52)	*HI*: 6.38 (2.33)<br>*MS*: 7.67 (1.97)<br>*PO*: 7.50 (1.78)<br>*HR*: 7.56 (1.24)	HI: 63<br>MS: 83<br>PO: 67<br>HR: 89	Yes	7.60 (0.48)	*HI*: 6.89 (0.78)<br>*MS*: 7.71 (0.76)<br>*PO*: 8.22 (0.67)<br>*HR*: 7.56 (1.24)	HI: 67<br>MS: 100<br>PO: 100<br>HR: 89	Yes	Yes
Health care equity independent of the region	7.07 (0.32)	*HI*: 6.88 (1.36)<br>*MS*: 7.17 (0.75)<br>*PO*: 7.55 (1.57)	HI: 75<br>MS: 83<br>PO: 73<br>HR: 70	Yes	7.12 (0.42)	*HI*: 6.44 (1.24)<br>*MS*: 7.14 (0.69)<br>*PO*: 7.33 (1.32)	HI: 67<br>MS: 86<br>PO: 78<br>HR: 89	Yes	Yes

**Delphi II/ Delphi I***	**Delphi I**<br><br>M_{total} (SD_{total})	**Delphi I**<br><br>M_{group} (SD_{group})	**Delphi I**<br><br>**Outcome "Critically important" (%)**	**Delphi I**<br><br>**Outcome included**	**Delphi II**<br><br>M_{total} (SD_{total})	**Delphi II**<br><br>M_{group} (SD_{group})	**Delphi II**<br><br>**Outcome "Critically important" (%)**	**Delphi II**<br><br>**Outcome included**	**COS**<br><br>**Outcome included**
		HR: 6.70 (2.06)				*HR*: 7.56 (1.01)			
Health care equity independent of patients' sociodemographic factors	7.48 (0.37)	*HI*: 6.88 (2.42)<br>*MS*: 7.83 (0.98)<br>*PO*: 7.50 (1.93)<br>*HR*: 7.70 (1.16)	*HI*: 75<br>*MS*: 100<br>*PO*: 75<br>*HR*: 80	Yes	7.48 (0.42)	*HI*: 7.00 (1.00)<br>*MS*: 7.14 (1.07)<br>*PO*: 7.78 (0.67)<br>*HR*: 8.00 (0.71)	*HI*: 78<br>*MS*: 86<br>*PO*: 100<br>*HR*: 100	Yes	Yes
**Outcomes related to the use of health care resources and non-health care resources**									
Outpatient/ regional providers (number)	6.20 (0.72)	HI: 6.00 (1.60)<br>MS: 5.50 (2.26)<br>PO: 7.42 (1.73)<br>HR: 5.90 (1.91)	HI: 50<br>MS: 33<br>PO: 58<br>HR: 40	No	6.30 (0.73)	*HI*: 6.00 (0.71)<br>*MS*: 5.86 (1.07)<br>*PO*: 7.56 (0.88)<br>*HR*: 5.78 (0.97)	HI: 22<br>MS: 14<br>PO: 89<br>HR: 33	No	No
Hospital beds (number)	4.71 (1.03)	HI: 3.38 (1.85)<br>MS: 4.17 (1.17)	HI: 13<br>MS: 0<br>PO: 42<br>HR: 30	No	5.31 (1.22)	*HI*: 3.44 (0.88)<br>*MS*: 5.00 (1.15)	HI: 0<br>MS: 14<br>PO: 33<br>HR: 67	No	No

**Delphi II/ Delphi I***	**Delphi I**<br><br>***M**_{total}* **(*SD*_{total})**	**Delphi I**<br><br>***M*_{group} *(SD_{group})***	**Delphi I**<br><br>**Outcome "Critically important" (%)**	**Delphi I**<br><br>**Outcome included**	**Delphi II**<br><br>***M**_{total}* **(*SD*_{total})**	**Delphi II**<br><br>***M*_{group} *(SD_{group})***	**Delphi II**<br><br>**Outcome "Critically important" (%)**	**Delphi II**<br><br>**Outcome included**	**COS**<br><br>**Outcome included**
		PO: 6.08 (2.68)<br>HR: 5.20 (2.15)				*PO*: 6.22 (0.67)<br>*HR*: 6.56 (1.42)			
Costs for the healthcare system	6.29 (0.62)	HI: 5.75 (1.67)<br>MS: 5.83 (1.72)<br>PO: 6.27 (2.00)<br>HR: 7.30 (1.49)	HI: 25<br>MS: 33<br>PO: 36<br>HR: 80	No	6.23 (0.40)	*HI*: 6.11 (0.93)<br>*MS*: 6.14 (0.69)<br>*PO*: 5.78 (1.20)<br>*HR*: 6.22 (2.11)	HI: 44<br>MS: 29<br>PO: 22<br>HR: 63	No	No
Cost efficiency of the healthcare system	6.45 (0.11)	*HI*: 6.38 (2.20)<br>*MS*: 6.50 (2.43)<br>*PO*: 6.33 (1.61)<br>*HR*: 6.60 (2.01)	HI: 50<br>MS: 50<br>PO: 33<br>HR: 50	No	6.53 (0.23)	*HI*: 6.33 (1.00)<br>*MS*: 6.57 (0.53)<br>*PO*: 6.33 (0.87)<br>*HR*: 6.89 (1.36)	HI: 44<br>MS: 57<br>PO: 33<br>HR: 78	No	No
Hospitals (number)	5.63 (1.01)	HI: 4.13 (1.55)<br>MS: 5.33 (1.51)	HI: 0<br>MS: 17<br>PO: 25<br>HR: 70	No	5.57 (0.88)	*HI*: 4.67 (1.00)<br>*MS*: 4.86 (0.69)	HI: 0<br>MS: 0<br>PO: 22<br>HR: 63	No	No

**Delphi II/ Delphi I***	**Delphi I**<br><br>***M*** M_{total} (SD_{total})	**Delphi I**<br><br>M_{group} (SD_{group})	**Delphi I**<br><br>**Outcome "Critically important" (%)**	**Delphi I**<br><br>**Outcome included**	**Delphi II**<br><br>M_{total} (SD_{total})	**Delphi II**<br><br>M_{group} (SD_{group})	**Delphi II**<br><br>**Outcome "Critically important" (%)**	**Delphi II**<br><br>**Outcome included**	**COS**<br><br>**Outcome included**
		PO: 6.25 (1.76)<br>HR: 6.80 (1.99)				*PO*: 5.89 (0.93)<br>*HR*: 6.22 (2.17)			
Staffing levels	7.65 (0.59)	HI: 6.88 (2.59)<br>MS: 8.33 (0.82)<br>PO: 8.09 (0.83)<br>HR: 7.30 (1.89)	HI: 75<br>MS: 100<br>PO: 100<br>HR: 70	Yes	7.77 (0.59)	*HI*: 6.89 (1.27)<br>*MS*: 8.29 (0.49)<br>*PO*: 8.33 (0.50)<br>*HR*: 7.56 (1.01)	HI: 78<br>MS: 100<br>PO: 100<br>HR: 89	Yes	Yes
Technical equipment of the inpatient providers	7.30 (0.65)	HI: 6.75 (1.39)<br>MS: 6.80 (1.48)<br>PO: 8.36 (0.81)<br>HR: 7.30 (1.42)	HI: 63<br>MS: 60<br>PO: 100<br>HR: 80	Yes	7.57 (0.52)	*HI*: 6.78 (0.44)<br>*MS*: 7.71 (0.95)<br>*PO*: 8.22 (0.67)<br>*HR*: 7.56 (0.88)	HI: 78<br>MS: 100<br>PO: 100<br>HR: 100	Yes	Yes
Visitors for patients	4.81 (0.67)	HI: 3.88 (2.03)<br>MS: 4.50 (1.87)	HI: 13<br>MS: 17<br>PO: 25<br>HR: 30	No	4.73 (0.69)	*HI*: 3.67 (1.22)<br>*MS*: 4.57 (0.53)	HI: 0<br>MS: 0<br>PO: 11<br>HR: 33	No	No

Delphi II/ Delphi I*	Delphi I<br><br>*M*$_{total}$ (*SD*$_{total}$)	Delphi I<br><br>*M*$_{group}$ (*SD*$_{group}$)	Delphi I<br><br>Outcome "Critically important" (%)	Delphi I<br><br>Outcome included	Delphi II<br><br>*M*$_{total}$ (*SD*$_{total}$)	Delphi II<br><br>*M*$_{group}$ (*SD*$_{group}$)	Delphi II<br><br>Outcome "Critically important" (%)	Delphi II<br><br>Outcome included	COS<br><br>Outcome included
		PO: 5.58 (1.62)<br>HR: 5.30 (1.95)				*PO*: 5.22 (1.09)<br>*HR*: 5.44 (2.30)			
Accommodation options for family/ relatives close to hospitals	5.01 (0.96)	*HI*: 4.00 (1.31)<br>*MS*: 4.67 (0.82)<br>*PO*: 6.58 (2.23)<br>*HR*: 4.80 (1.62)	HI: 0<br>MS: 0<br>PO: 67<br>HR: 20	No	4.84 (1.03)	*HI*: 3.67 (1.12)<br>*MS*: 4.14 (1.07)<br>*PO*: 6.33 (0.87)<br>*HR*: 5.22 (1.86)	HI: 0<br>MS: 0<br>PO: 56<br>HR: 11	No	No
**Health care provider outcomes**									
Employee workload	6.86 (0.68)	HI: 5.75 (1.91)<br>MS: 7.00 (1.10)<br>PO: 7.58 (1.38)<br>HR: 7.10 (1.45)	HI: 50<br>MS: 83<br>PO: 75<br>HR: 60	Yes	7.16 (0.48)	*HI*: 6.56 (0.73)<br>*MS*: 6.86 (1.21)<br>*PO*: 7.78 (0.83)<br>*HR*: 7.44 (1.59)	HI: 44<br>MS: 71<br>PO: 100<br>HR: 78	Yes	Yes
Employee turnover	6.05 (0.77)	HI: 6.00 (1.07)	HI: 25<br>MS: 17<br>PO: 83	No	6.33 (0.44)	*HI*: 6.00 (1.22)	HI: 33<br>MS: 57<br>PO: 78	No	No

**Delphi II/ Delphi I***	**Delphi I**<br><br>***M**_{total}* **(*SD*_{total})**	**Delphi I**<br><br>***M*_{group} (*SD_{group}*)**	**Delphi I**<br><br>**Outcome "Critically important" (%)**	**Delphi I**<br><br>**Outcome included**	**Delphi II**<br><br>***M_{total}* (*SD*_{total})**	**Delphi II**<br><br>***M*_{group} (*SD_{group}*)**	**Delphi II**<br><br>**Outcome "Critically important" (%)**	**Delphi II**<br><br>**Outcome included**	**COS**<br><br>**Outcome included**
		MS: 5.83 (1.94)<br>PO: 7.25 (1.36)<br>HR: 5.10 (2.08)	HR: 10			*MS*: 6.43 (1.13)<br>*PO*: 7.00 (0.71)<br>*HR*: 5.89 (1.62)	HR: 33		
Employee job satisfaction	6.88 (0.59)	HI: 5.88 (1.81)<br>MS: 7.00 (7.00)<br>PO: 7.33 (1.30)<br>HR: 7.30 (1.16)	HI: 38<br>MS: 67<br>PO: 75<br>HR: 70	No	7.01 (0.46)	*HI*: 6.22 (0.83)<br>*MS*: 7.14 (1.07)<br>*PO*: 7.33 (0.87)<br>*HR*: 7.33 (1.32)	HI: 22<br>MS: 86<br>PO: 89<br>HR: 78	Yes	Yes
Training positions for junior physicians (quality, number)	6.67 (0.72)	HI: 5.63 (1.41)<br>MS: 7.50 (1.05)<br>PO: 7.17 (1.64)<br>HR: 6.40 (2.41)	HI: 38<br>MS: 83<br>PO: 75<br>HR: 60	Yes	6.82 (0.61)	*HI*: 6.00 (0.71)<br>*MS*: 7.71 (0.76)<br>*PO*: 6.89 (1.27)<br>*HR*: 6.67 (1.50)	HI: 22<br>MS: 100<br>PO: 78<br>HR: 67	Yes	Yes
Routine/ experience of members of the	7.88 (0.67)	HI: 8.75 (0.71)	HI: 100<br>MS: 67	Yes	7.90 (0.41)	*HI*: 7.89 (1.05)	HI: 89<br>MS: 100	Yes	Yes

**Delphi II/ Delphi I***	**Delphi I**<br><br>***M*_total_ (*SD*_total_)**	**Delphi I**<br><br>***M*_group_ (*SD_group_*)**	**Delphi I**<br><br>**Outcome "Critically important" (%)**	**Delphi I**<br><br>**Outcome included**	**Delphi II**<br><br>***M*_total_ (*SD*_total_)**	**Delphi II**<br><br>***M*_group_ (*SD_group_*)**	**Delphi II**<br><br>**Outcome "Critically important" (%)**	**Delphi II**<br><br>**Outcome included**	**COS**<br><br>**Outcome included**
treatment team (number of cases)/ Routine of members of the treatment team (number of cases)*		MS: 7.00 (2.10)<br>PO: 8.25 (0.97)<br>HR: 7.50 (1.18)	PO: 92<br>HR: 90			*MS*: 7.71 (0.76)<br>*PO*: 8.56 (0.53)<br>*HR*: 7.44 (1.24)	PO: 100<br>HR: 89		
Routine/ experience of surgeons (number of cases)/ Routine of surgeons (number of cases)*	8.15 (0.61)	HI: 8.88 (0.35)<br>MS: 7.33 (1.75)<br>PO: 8.78 (0.90)<br>HR: 7.80 (0.79)	HI: 100<br>MS: 83<br>PO: 92<br>HR: 100	Yes	8.23 (0.50)	*HI*: 8.56 (0.53)<br>*MS*: 7.71 (0.95)<br>*PO*: 8.89 (0.33)<br>*HR*: 7.78 (1.30)	HI: 100<br>MS: 100<br>PO: 100<br>HR: 89	Yes	Yes
**Diverse outcomes**									
Shifts in the provision of hospital services	6.46 (0.63)	HI: 5.86 (1.68)<br>MS: 7.00 (1.55)<br>PO: 7.17 (1.27)<br>HR: 5.80 (1.62)	HI: 25<br>MS: 83<br>PO: 83<br>HR: 30	Yes	6.85 (0.83)	*HI*: 5.89 (0.93)<br>*MS*: 7.00 (0.82)<br>*PO*: 6.75 (1.16)<br>*HR*: 5.67 (1.22)	HI: 11<br>MS: 71<br>PO: 75<br>HR: 22	No	No

Delphi II/ Delphi I*	Delphi I<br><br>M_{total} (SD_{total})	Delphi I<br><br>M_{group} (SD_{group})	Delphi I<br><br>Outcome "Critically important" (%)	Delphi I<br><br>Outcome included	Delphi II<br><br>M_{total} (SD_{total})	Delphi II<br><br>M_{group} (SD_{group})	Delphi II<br><br>Outcome "Critically important" (%)	Delphi II<br><br>Outcome included	COS<br><br>Outcome included
Co-operations, networking between health service providers/ Co-operations between health service providers*	7.39 (0.45)	HI: 6.63 (0.74)<br>MS: 7.50 (1.38)<br>PO: 7.75 (1.29)<br>HR: 7.70 (1.34)	HI: 50<br>MS: 67<br>PO: 83<br>HR: 80	Yes	7.58 (0.37)	*HI*: 7.00 (1.58)<br>*MS*: 7.57 (0.79)<br>*PO*: 8.00 (1.00)<br>*HR*: 7.75 (1.28)	HI: 67<br>MS: 100<br>PO: 89<br>HR: 88	Yes	Yes
Population's trust in healthcare/ Psychological safety of the population with respect to healthcare*	6.00 (0.60)	HI: 5.00 (2.33)<br>MS: 6.50 (1.87)<br>PO: 6.11 (2.32)<br>HR: 6.40 (2.55)	HI: 13<br>MS: 50<br>PO: 56<br>HR: 50	No	6.78 (0.34)	*HI*: 6.22 (1.56)<br>*MS*: 7.00 (1.15)<br>*PO*: 6.78 (1.56)<br>*HR*: 7.11 (1.69)	HI: 33<br>MS: 57<br>PO: 56<br>HR: 56	No	No
Outpatient care physicians' knowledge about healthcare	7.29 (0.10)	HI: 7.13 (1.25)<br>MS: 7.33 (1.75)<br>PO: 7.30 (1.57)<br>HR: 7.40 (1.78)	HI: 75<br>MS: 83<br>PO: 70<br>HR: 80	Yes	7.31 (0.24)	*HI*: 7.67 (0.71)<br>*MS*: 7.00 (0.82)<br>*PO*: 7.33 (1.22)<br>*HR*: 7.22 (1.20)	HI: 100<br>MS: 71<br>PO: 78<br>HR: 78	Yes	Yes

Delphi II/ Delphi I*	Delphi I<br><br>*M*M_{total} (SD_{total})	Delphi I<br><br>M_{group} (SD_{group})	Delphi I<br><br>Outcome "Critically important" (%)	Delphi I<br><br>Outcome included	Delphi II<br><br>M_{total} (SD_{total})	Delphi II<br><br>M_{group} (SD_{group})	Delphi II<br><br>Outcome "Critically important" (%)	Delphi II<br><br>Outcome included	COS<br><br>Outcome included
Patients' knowledge about healthcare	6.78 (0.55)	HI: 7.50 (0.76)<br>MS: 6.00 (1.79)<br>PO: 6.64 (1.69)<br>HR: 7.00 (2.21)	HI: 100<br>MS: 50<br>PO: 64<br>HR: 70	No	6.84 (0.23)	*HI*: 6.67 (0.71)<br>*MS*: 7.00 (1.00)<br>*PO*: 7.13 (1.36)<br>*HR*: 6.56 (1.67)	HI: 56<br>MS: 57<br>PO: 75<br>HR: 44	No	No
Transparency (e.g. of treatment quality and clinical pathways)	7.33 (0.51)	HI: 7.63 (2.07)<br>MS: 7.00 (1.79)<br>PO: 8.00 (0.85)<br>HR: 6.70 (1.83)	HI: 88<br>MS: 67<br>PO: 100<br>HR: 60	Yes	7.32 (0.62)	*HI*: 7.56 (1.01)<br>*MS*: 6.71 (0.95)<br>*PO*: 8.22 (0.67)<br>*HR*: 6.78 (1.72)	HI: 78<br>MS: 43<br>PO: 100<br>HR: 56	Yes	Yes

*HI = Representatives of statutory health insurances, MS = Representatives of medical societies, PO = Representatives of patient organizations, HR = Health Services Researchers
